# Highly *Z*-Selective Horner–Wadsworth–Emmons Olefination Using Modified Still–Gennari-Type Reagents

**DOI:** 10.3390/molecules27207138

**Published:** 2022-10-21

**Authors:** Ignacy Janicki, Piotr Kiełbasiński

**Affiliations:** Division of Organic Chemistry, Centre of Molecular and Macromolecular Studies, Polish Academy of Sciences, Sienkiewicza 112, 90-363 Łódź, Poland

**Keywords:** HWE reaction, Still-Gennari olefination, Ando olefination, stereoselective synthesis, *Z*-selectivity, Wittig reaction, phosphonates, alkenes, hexafluoroisopropanol, C=C bond formation

## Abstract

In this report, new, easily accessible reagents for highly *Z*-selective HWE reactions are presented. Alkyl di-(1,1,1,3,3,3-hexafluoroisopropyl)phosphonoacetates, structurally similar to Still–Gennari type reagents, were tested in HWE reactions with a series of various aldehydes. Very good *Z*-selectivity (up to a 98:2 *Z*:*E* ratio) was achieved in most cases along with high yields. Application of the new reagents may be a valuable, practical alternative to the well-established Still–Gennari or Ando *Z*-selective carbonyl group olefination protocols.

## 1. Introduction

**Scheme 1:**
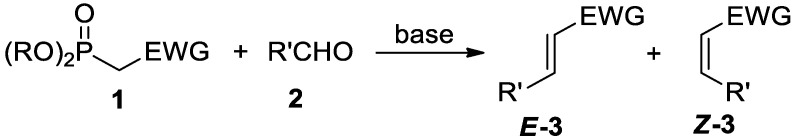
General scheme of the Horner–Wadsworth–Emmons reaction.

**Figure 1:**
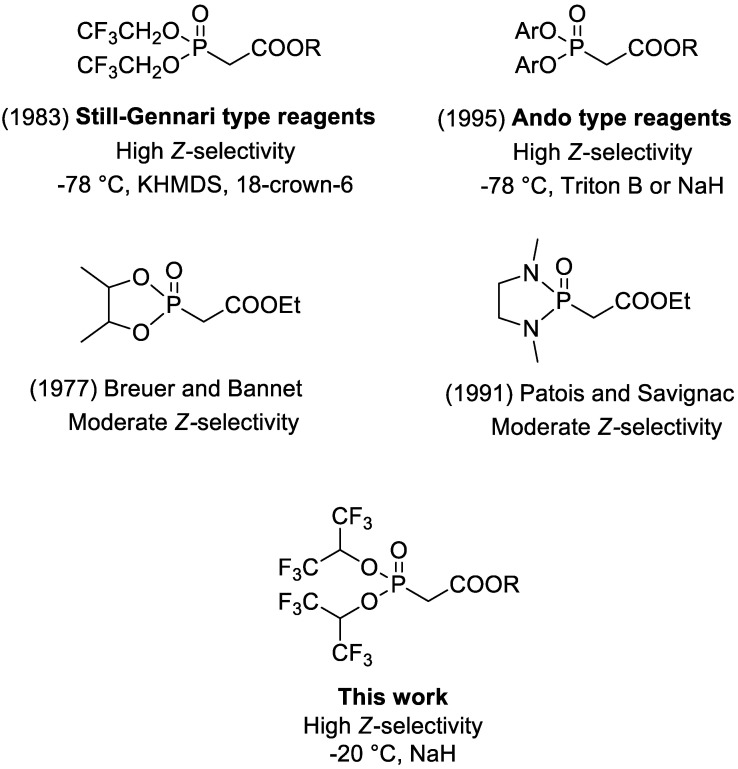
Comparison of *Z*-selective reagents for the HWE reaction [11,12,13,14].

Stereoselective alkene synthesis is one of the major challenges in organic synthesis [1]. The configuration of carbon–carbon double bonds affects all properties of molecules, therefore, highly selective methods for the synthesis of *E* or *Z* olefins are of great value. However, *Z*-selective reactions are considerably more difficult and less developed, mainly because of thermodynamic factors, which usually favor formation of the more stable *E*-products [2]. One of the well-established, typically highly *E*-selective alkene formation methods is the Horner–Wadsworth–Emmons (HWE) reaction, which is based on the olefination of carbonyl groups using dialkyl phosphonate reagents (Scheme 1) [3,4,5,6,7]. Its high *E*-selectivity results from the thermodynamic stabilization of *E*-products and intermediates leading to its formation. The selectivity of the HWE reaction is one of its important advantages, but in its classical form, it is restricted to the synthesis of *E*-alkenes. Nevertheless, the selectivity of the HWE reaction is highly dependent on the structure of the phosphonate reagents and it can be modified [8,9]. Attempts to develop *Z*-selective HWE reagents were made already in the late 1970s [10,11,12], but the first reliable and highly *Z*-selective modification of HWE reaction was reported in 1983 by Still and Gennari (Figure 1) [13]. In the standard HWE reaction, diethyl or dimethyl phosphonate reagents are usually applied. The Still–Gennari modification of the HWE reaction, fairly called “Still–Gennari olefination” due to its broad applicability and inverted selectivity, is based on the application of bis(2,2,2-trifluoroethyl) phosphonate reagents for the olefination of carbonyl compounds, usually in the presence of a strong base system—potassium bis(trimethylsilyl)amide (KHMDS) with 18-crown-6 crown ether. Along with the modification developed in the mid-1990s by Ando [14,15,16,17,18], the Still–Gennari olefination is one of the most widely applied *Z*-selective modifications of the HWE reaction. Its scope of applications was recently discussed in our review article [19].

Still–Gennari and Ando-type reagents constitute important tools for the *Z*-selective alkene formation. However, examples from a total synthesis of biologically active, complex molecules show that achieving high *Z*-selectivity using these reagents is not always easy and the outcome of the olefination reactions is highly dependent on the reaction conditions and the type of reagent used [19]. Therefore, it would be desirable to broaden the scope of reliable *Z*-selective carbonyl olefination reagents in order to improve our synthetic toolbox.

The *Z*-selectivity of Still–Gennari olefination is a result of the kinetic control of the reaction. An electron-withdrawing effect of R groups (Scheme 1), such as 2,2,2-trifluoroethyl or phenyl, favors the *Z*-selective course of the reaction in contrast to standard *E*-selective HWE reaction where R is usually the ethyl group (pK_a_ of 2,2,2-trifluoroethanol is 12.4 and pK_a_ of phenol is 10, while pK_a_ of ethanol is 16). The correlation between the electron-withdrawing effect of the R group and the stereoselectivity of the reaction was investigated in more detail by Motoyoshiya and coworkers [9]. Moreover, steric hindrance of R groups may further affect *Z*-selectivity as in the case of Ando-type reagents bearing aryl substituents.

In our previous study, we reported a very simple protocol for the synthesis of Still–Gennari and Ando-type phosphonates [20]. We also reported the synthesis of new phosphonate reagents bearing 1,1,1,3,3,3-hexafluoroisopropyl R groups. These compounds are expected to be highly *Z*-selective olefination reagents because of a stronger electron-withdrawing effect of 1,1,1,3,3,3-hexafluoroisopropyl R groups (pK_a_ of 1,1,1,3,3,3-hexafluoroisopropanol is 9.4). In the present research, we decided to test the performance of our new reagents and evaluate their applicability on the basis of a series of model reactions with various aldehydes.

## 2. Results and Discussion

**Scheme 2:**
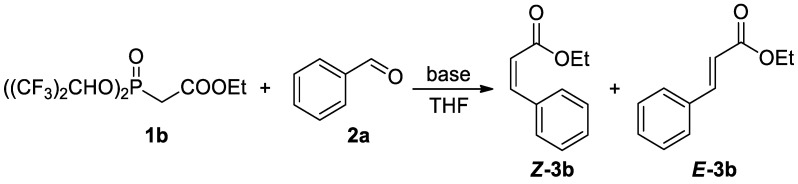
Optimization of conditions based on the reaction of **1b** with benzaldehyde **2a**.

The reagents being subjects of this study, methyl, and ethyl bis(1,1,1,3,3,3-hexafluoroisopropyl) phosphonates **1a** and **1b**, were synthesized according to our previously reported procedure [20]. Because of a structural resemblance and similar reactivity we may consider **1a** and **1b** as “Still–Gennari-type” reagents. We decided to test these reagents for the synthesis of disubstituted alkenes by *Z*-selective HWE reaction. In order to maximize the yield and the stereoselectivity of the reaction, optimization of the reaction conditions was necessary (Table 1, Scheme 2).

During the optimization study, several base systems were evaluated in the reaction of **1b** with benzaldehyde at various temperatures (Scheme 2). All the reactions were run in THF for 1 h. When using NaH as a base at −78 °C, the reaction was very slow, only traces of *Z*-product could be detected after 1 h (Table 1, entry 1). This is most probably due to the slow deprotonation of the phosphonate reagent at this low temperature because when the reaction was heated from −78 °C to room temperature, hydrogen gas evolved, and the reaction proceeded. Based on this observation, higher temperatures were evaluated (Table 1, entries 2–4). Quite unexpectedly, the best results regarding yield (94%) and selectivity (97:3 *Z*:*E*) were obtained using NaH at −20 °C (Table 1, entry 3), while Still–Gennari olefination is usually conducted at lower temperatures (typically −78 °C). This result is very promising because it shows that by using our new reagents high stereoselectivity may be achieved at higher temperatures. Only a slight decrease in stereoselectivity was observed at 0 °C (Table 1, entry 4). It is noteworthy that using an excess of a base resulted in a significant decrease in the yield (Table 1, entry 5). The possibility of increasing the stereoselectivity of the reaction by providing additional sodium ions to the reaction mixture according to Pihko et al. was also investigated. However, no influence of the additive on the reaction course was observed (Table 1, entry 6) [21].

In contrast to the classic Still–Gennari olefination protocol, application of KHMDS or KHMDS with 18-crown-6 additive appears to be an inferior option (we made a similar observation in our previous work concerning the synthesis of *Z*-α,β-unsaturated phosphonates) [22]. The yields of the reactions were moderate (34–61%) and the selectivity was lower in comparison to the results obtained with NaH (up to a 91:9 *Z*:*E* ratio—Table 1, entries 7–10). Running the reaction at a lower temperature somewhat favored *Z*-selectivity, however, it decreased the yield (Table 1, entries 8 and 10). The addition of crown ether surprisingly decreased the selectivity of the reaction. Moreover, in our hands, the reaction with KHMDS tends to be a little capricious, sensitive to the reaction conditions, and difficult to reproduce, since we have previously reported better results which we were unable to repeat now.

**Scheme 3:**
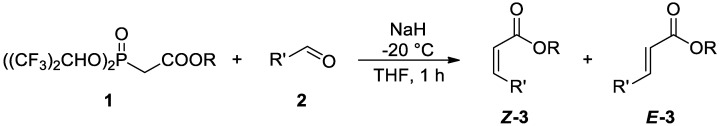
Reaction of reagents **1** with various aldehydes **2** under optimized conditions.

Other bases which were tested include *t*-BuOK, K_2_CO_3_, triton-B (benzyltrimethylammonium hydroxide), and (CF_3_)_2_CHONa. Reaction with *t*-BuOK at −20 °C gave 62% yield of the product in only an 81:19 *Z*:*E* ratio (Table 1, entry 11), however, conducting the reaction at a lower temperature may improve the yield and *Z*-selectivity to 80% and 92:8 *Z*:*E*, as presented earlier [20]. Unfortunately, the reaction with a mild base K_2_CO_3_ was unsuccessful, and only traces of *Z*-product were detected (Table 1, entry 12). Interestingly, the application of triton-B (according to Ando) [14] inverted the stereoselectivity of the reaction (14:86 *Z*:*E* ratio) proving the high influence of the reaction conditions on the observed results (Table 1, entry 13). Unexpectedly, very good results were obtained using (CF_3_)_2_CHONa—93% yield and 96:4 *Z*:*E* product ratio. Based on this observation we decided to investigate the possibility of using this base with other HWE reagents (Table 2, Scheme 3).

The application of (CF_3_)_2_CHONa as a base in the standard HWE reaction of methyl dimethylphosphonoacetate **1c** or ethyl diethylphosphonoacetate **1d** with benzaldehyde resulted in excellent *E*-selectivity and very good yields (Table 2, entries 1 and 2). This observation indicates that (CF_3_)_2_CHONa may be successfully used in HWE reactions. Despite excellent results with **1b** and standard HWE reagents **1c** and **1d**, the reaction using (CF_3_)_2_CHONa with Still–Gennari and Ando-type phosphonates (**1e** and **1f,** respectively) was only moderately stereoselective, although very high yielding (Table 2, entries 3–6). It is noteworthy that lowering the temperature to −78 °C resulted in little increased *Z*-selectivity compared to the reaction at −20 °C, without significant loss of yield.

**Figure 2:**
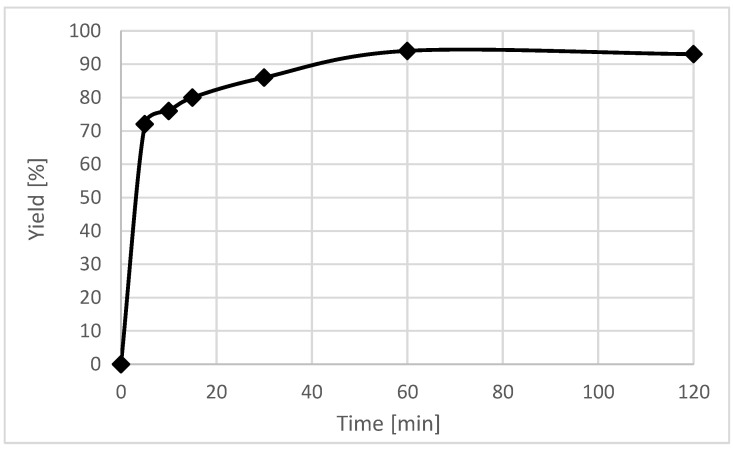
Time course of the reaction of **1b** and benzaldehyde with NaH at −20 °C.

The time course of the reaction of **1b** with benzaldehyde in the presence of NaH at −20 °C was also investigated (Figure 2). Measurements were taken after 5, 10, 15, 30, 60, and 120 min. The reaction proceeded very fast, after 5 min the yield reached 72% and the reaction was complete within 1 h (94%).

Based on the above observations, all further reactions of **1a** and **1b** with a series of various aldehydes **2a**–**2m** were carried out in THF at −20 °C for 1 h, using NaH as a base (Scheme 3, Table 3). Generally, similar reaction yields were observed for both reagents **1a** and **1b**; however, slightly better stereoselectivities were observed for ethyl bis-(1,1,1,3,3,3-hexafluoroisopropyl)phosphonoacetate **1b** than for methyl bis-(1,1,1,3,3,3-hexafluoroisopropyl)phosphonoacetate **1a**.

Very good results were obtained with most of the aromatic aldehydes tested (Table 3, entries 1–9). The standard reaction of **1a** or **1b** with benzaldehyde **2a** gave very high yields of products **3aa** and **3ba** respectively along with excellent *Z*-selectivity with a 97:3 *Z*:*E* ratio. Similarly, reactions with *para*, *meta*, and *ortho* tolualdehydes **2b**–**2d** resulted in high yields of products **3ab**–**3ad** and **3bb**–**3bd** (81–87%) and a very high *Z*-selectivity. Besides minimally better stereoselectivity with *o*-tolualdehyde **2d**, no significant differences in reactivity of **2b**–**2d** with **1a** or **1b** were observed. Olefination of *para* chloro, bromo, and nitro benzaldehydes **2e**, **2f**, and **2g**, respectively, proceeds in a nearly quantitative manner (93–99% yield) with very high *Z*-selectivity as well (94:6–96:4 *Z*:*E* ratio). Olefination of heterocyclic furfural **2h** and 2-thiophenecarboxaldehyde **2i** resulted in slightly lower yields, however, the reactions were still highly stereoselective.

Olefination of α,β-unsaturated cinnamaldehyde **2j** and aliphatic aldehydes **2k**–**2m** using reagents **1a** and **1b** gave the corresponding products **3aj**–**3am** and **3bj**–**3bm** in good yields (69–90%) with high *Z*-selectivity (86:14–91:9 *Z*:*E* ratio, Table 3, entries 10–13). Similar to that reported for Ando and Still–Gennari *Z*-selective HWE reaction, olefination of aliphatic aldehydes resulted in a bit inferior selectivity compared to reactions with aromatic aldehydes. Nevertheless, the results obtained with our new reagents **1a** and **1b** are comparable with those previously reported [13,14,15,16,17,18].

In order to compare the performance of the newly developed reagent **1b** under the reported conditions (using NaH in dry THF at −20 °C) with standard Still–Gennari reagent **1e**, two model reactions with benzaldehyde and octanal were performed (Table 3, entries 1 and 12 in brackets). The *Z:E* selectivity using Still–Gennari reagent **1e** with NaH at −20 °C was found to be inferior both in the olefination of aromatic and aliphatic aldehydes. The reaction of **1e** with benzaldehyde resulted in a quantitative yield but only moderate *Z*:*E* selectivity 74:26, while the application of **1b** resulted in an excellent 97:3 *Z*:*E* ratio. Similarly, the reaction of **1e** with octanal resulted in poorer *Z*:*E* selectivity (78:22) than the reaction using reagent **1b** (88:12 *Z*:*E* ratio). These observations (along with data from Table 1—entries 2–4) suggest that a very good stereochemical outcome of the reactions with **1b** may be achieved using an easily accessible base, at higher temperatures than in the case of Still–Gennari reagent **1e**, as typically −78 °C and KHMDS with 18-crown-6 additive is required in order to achieve high *Z*-selectivity in standard Still–Gennari olefination.

## 3. Materials and Methods

### 3.1. General Information

The NMR spectra were recorded using a Bruker Avance Neo 400 spectrometer. Dimethyl terephthalate was used as an internal standard in all NMR experiments [23]. All solvents were dried and distilled prior to use. All the starting materials were purchased from Merck, Sigma-Aldrich, TCI Chemicals, or Fluorochem. Reagents **1a** and **1b** were prepared according to the procedure reported earlier [20]. All the reactions were run in duplicate. The spectra of all the products obtained were in agreement with the data reported in the literature (see Supplementary Materials) [24,25,26,27,28,29,30,31,32,33,34,35,36,37,38].

### 3.2. General Procedure for the Reaction of ***1a*** or ***1b*** with Aldehydes ***2a*–*2m***

In a round bottom flask with a magnetic stirrer, under an argon atmosphere, 1.2 mmol of base (typically sodium hydride—48 mg of 60% dispersion in mineral oil) was placed and 3 mL of THF was added. The solution was cooled to −20 °C and 1.3 mmol of reagent **1a** (590 mg) or **1b** (608 mg) in 2 mL of dry THF was added. The reaction mixture was stirred for 15 min and 1 mmol of appropriate aldehyde in 5 mL of THF was added. After 1 h, 0.5 mL samples were collected by a syringe and quenched with saturated NH_4_Cl solution. The aqueous layer was extracted two times with 0.5 mL of CH_2_Cl_2_. Combined organic fractions were dried using anhydrous Na_2_SO_4_, filtered, and next condensed under reduced pressure. To a thus obtained crude product, a specific amount of dimethyl terephthalate was added (as an internal standard for the ^1^H-NMR measurements), and the mixture was dissolved in CDCl_3_ to take ^1^H-NMR spectra. Yields and *Z*:*E* product ratios of the reactions were calculated based on ^1^H-NMR with an internal standard [12].

## 4. Conclusions

In conclusion, we have developed a successful application of new reagents, methyl, and ethyl bis(1,1,1,3,3,3-hexafluoroisopropyl)phosphonates, **1a** and **1b** in a highly *Z*-selective HWE reaction. The reagents are easily accessible via previously reported synthetic protocol [20]. In contrast to previous *Z*-selective HWE reagents, the application of **1a** or **1b** does not require very low temperatures (−78 °C) to achieve high stereoselectivity. Moreover, readily accessible sodium hydride was found to be a very good base for the presented reaction.

Olefination of aromatic aldehydes using reagents **1a** and **1b** gives excellent results—up to a 98:2 *Z*:*E* product ratio, and up to quantitative yield. Slightly lower, however, very high *Z*-selectivity can also be achieved in the olefination of aliphatic aldehydes. The presented reagents may constitute a valuable alternative to well-established Ando and Still–Gennari-type reagents for highly *Z*-selective olefination of carbonyl compounds, especially in the total synthesis of complex biologically active products.

## Figures and Tables

**Table 1:** Reaction of **1b** with benzaldehyde **2a**—optimization ^[a]^.

Entry	Base	Temperature	Yield ^[b]^	*Z*:*E* Ratio ^[c]^
1	NaH	−78 °C	traces	---
2	NaH	−40 °C	82%	97:3
3	NaH	−20 °C	94%	97:3
4	NaH	0 °C	85%	95:5
5	NaH (excess) ^[d]^	0 °C	55%	95:5
6	NaH + NaI ^[e]^	−20 °C	95%	97:3
7	KHMDS	−78 °C	37%	91:9
8	KHMDS + 18-crown-6 ^[f]^	−78 °C	34%	84:16
9	KHMDS	−40 °C	52%	90:10
10	KHMDS + 18-crown-6 ^[f]^	−40 °C	61%	86:14
11	*t*-BuOK	−20 °C	62%	81:19
12	K_2_CO_3_	r.t.	traces	traces of *Z*
13	Triton-B	−20 °C	23%	14:86
14	(CF_3_)_2_CHONa	−20 °C	93%	96:4

^[a]^ All the reactions were conducted in 10 mL of THF for 1 h by analogy with the general procedure (see Section 3.2); ^[b]^ yield was determined by ^1^H-NMR with dimethyl terephthalate as internal standard; ^[c]^ determined by ^1^H-NMR; ^[d]^ 0.15 equivalent excess of NaH was used; ^[e]^ 1 equivalent of NaI was used; ^[f]^ 5 equivalents of 18-crown-6 were used.

**Table 2:** HWE reaction of phosphonate reagents **1** and benzaldehyde **2a** with (CF_3_)_2_CHONa as a base ^[a]^.

Entry	Reagent	Temperature	Yield ^[b]^	*Z*:*E* Ratio ^[c]^
1	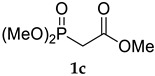	−20 °C	87%	2:98
2	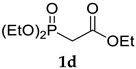	−20 °C	79%	>99% *E*
3	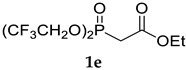	−20 °C	99%	68:32
4		−78 °C	96%	75:25
5	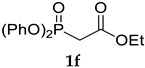	−20 °C	99%	64:36
6		−78 °C	97%	68:32

^[a]^ All the reactions were conducted in 10 mL of THF for 1 h according to the general procedure (see Section 3.2). ^[b]^ Yield was determined by ^1^H-NMR with dimethyl terephthalate as an internal standard. ^[c]^ Determined by ^1^H-NMR.

**Table 3:** Reactions of **1a** and **1b** with aldehydes **2a–2m** ^[a]^.

Entry	Substrate	Aldehyde	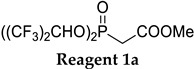			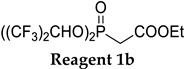		
			Product	Yield ^[b]^	*Z*:*E*^[c]^	Product	Yield ^[b]^	*Z*:*E*^[c]^
1	**2a**	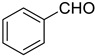	**3aa**	91%	97:3	**3ba**	95% (99%) ^[d]^	97:3 (74:26) ^[d]^
2	**2b**	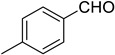	**3ab**	84%	96:4	**3bb**	81%	96:4
3	**2c**	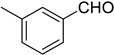	**3ac**	84%	95:5	**3bc**	86%	96:4
4	**2d**	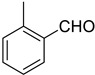	**3ad**	84%	97:3	**3bd**	87%	98:2
5	**2e**	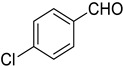	**3ae**	98%	95:5	**3be**	99%	96:4
6	**2f**	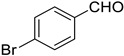	**3af**	95%	95:5	**3bf**	95%	94:6
7	**2g**	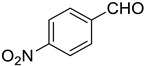	**3ag**	99%	94:6	**3bg**	93%	95:5
8	**2h**	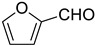	**3ah**	88%	92:8	**3bh**	79%	95:5
9	**2i**	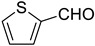	**3ai**	79%	94:6	**3bi**	89%	95:5
10	**2j**	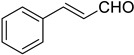	**3aj**	78%	89:11	**3bj**	82%	91:9
11	**2k**	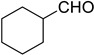	**3ak**	69%	86:14	**3bk**	70%	87:13
12	**2l**	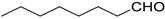	**3al**	88%	88:12	**3bl**	90% (92%) ^[d]^	88:12 (78:22) ^[d]^
13	**2m**	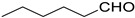	**3am**	77%	88:12	**3bm**	90%	88:12

^[a]^ All the reactions were conducted according to the general procedure (see Section 3.2). ^[b]^ Yield was determined by ^1^H-NMR with dimethyl terephthalate as the internal standard. ^[c]^ Determined by ^1^H-NMR. ^[d]^ Reaction with Still–Gennari reagent **1e**.

## Data Availability

Not applicable.

## Supplementary Materials

The following supporting information can be downloaded at: https://www.mdpi.com/article/10.3390/molecules27207138/s1, Section S1: Time study; Section S2: NMR spectra.
