# Supplementary material for: Highly Z-Selective Horner–Wadsworth–Emmons Olefination Using Modified Still–Gennari-Type Reagents
Source: Molecules. 2022 Oct 21;27(20):7138. doi: 10.3390/molecules27207138 (PMC9609750; doi:10.3390/molecules27207138)

Supporting information

"Highly *Z*-Selective Horner-Wadsworth-Emmons Olefination Using Modified Still-Gennari Type Reagents"

Author: Ignacy Janicki* and Piotr Kiełbasiński*

Table of contents

Time study 2

NMR spectra 3

Section S1. Time study

The reaction was conducted according to general procedure described in the main text. Yields were established by quantitative ^{1}H NMR measurements with dimethyl terephthalate as an internal standard.

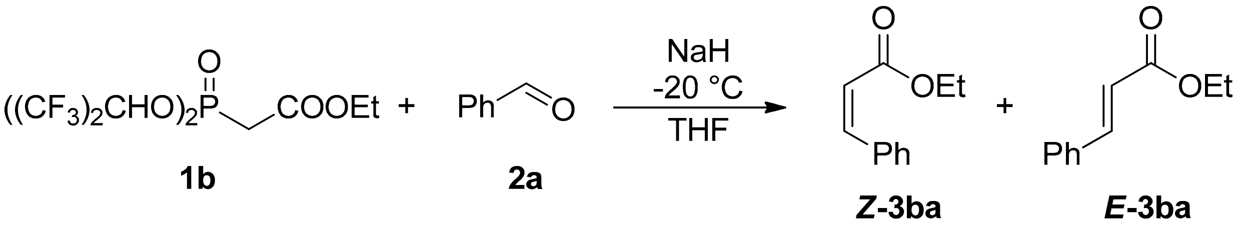

Scheme S1. Reaction of **1b** wih **2a** – time study.

Table S1. Reaction of **1b** wih **2a** – time study.

entry	time [min]	yield [%]
1	0	0
2	5	72
3	10	76
4	15	80
5	30	86
6	60	94
7	120	93

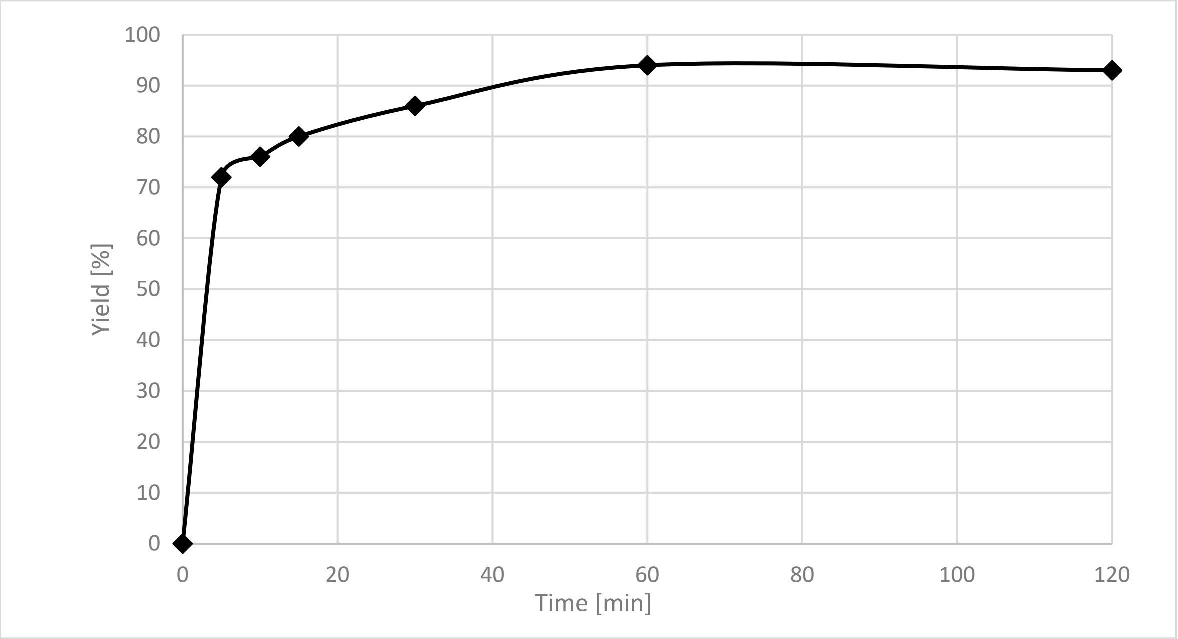

Figure S1. Reaction of **1b** wih **2a** – time study

Section S2. NMR spectra

The NMR spectra were recorded using a Bruker Avance Neo 400 spectrometer. All the products obtained were in agreement with the data reported in the literature,[20-24-38] therefore further chromatographic purification step was omitted and the reaction yield was calculated based on ^{1}H NMR spectra of raw extracted reaction mixture with a carefully weighed aliquot of internal standard.[23] Dimethyl terephthalate was used as an internal standard in all NMR experiments. Dimethyl terephthalate ^{1}H NMR (400 MHz, Chloroform-*d*) δ 8.10 (s, 4H), 3.94 (s, 6H). *Z:E* ratio was calculated based on *Z* and *E* products vinyl protons integration ratio.

1a

^{1}H NMR (400 MHz, Chloroform-*d*) δ 5.32 – 5.17 (m, 2H), 3.78 (s, 3H), 3.29 (d, *J* = 21.6 Hz, 2H). [20]

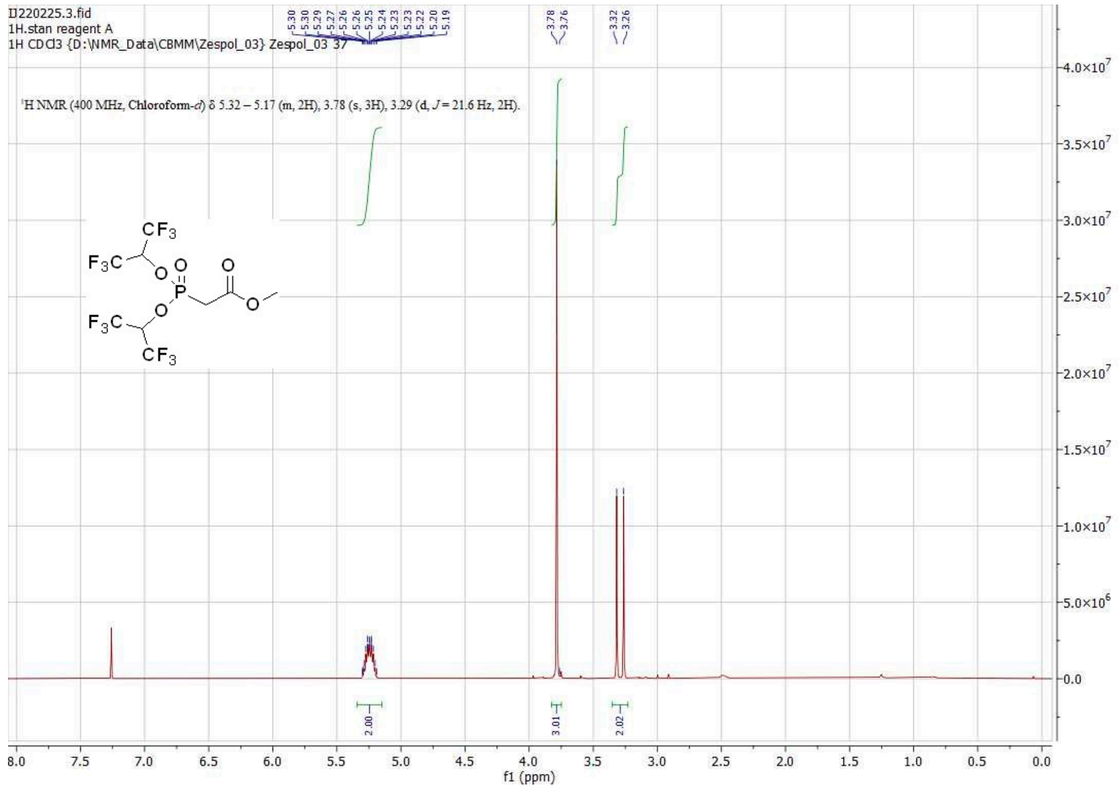

^{31}P NMR (162 MHz, Chloroform-*d*) δ 25.88. [20]

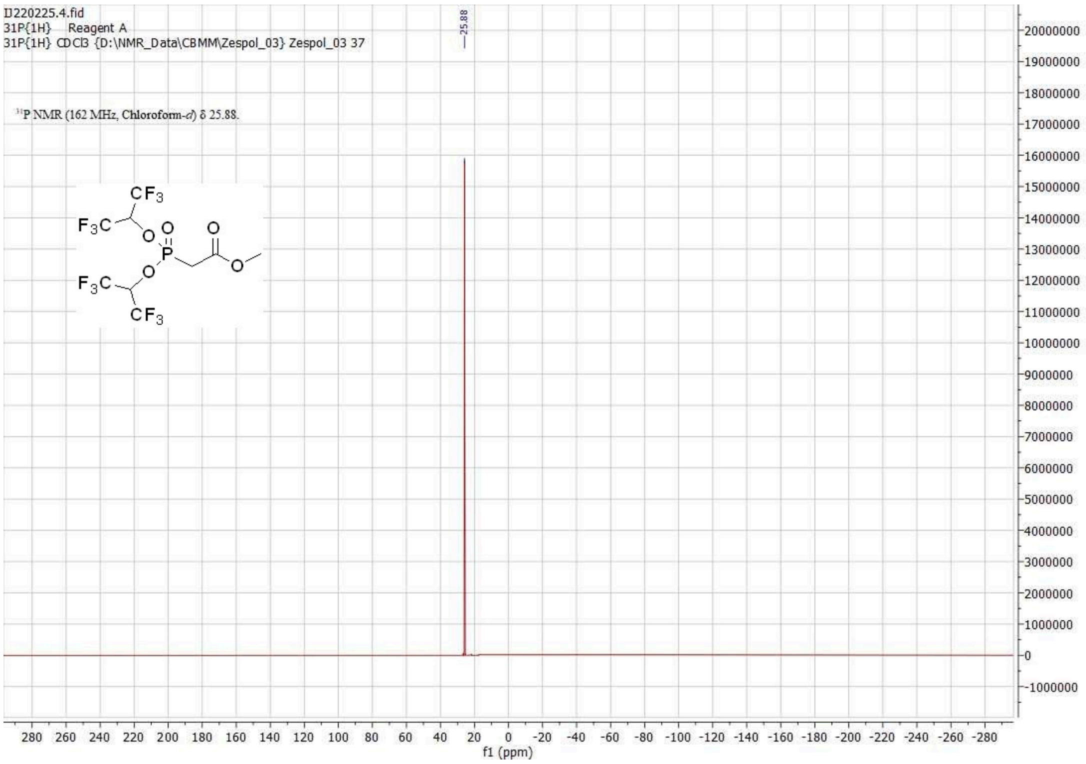

1b

^{1}H NMR ($CDCl_3$, 500 MHz): δ = 5.35-5.17 (m, 2H), 4.23 (q, *J* = 7.2 Hz, 2H), 3.27 (d, *J* = 21.6 Hz, 2H), 1.28 (t, *J* = 7.2 Hz, 3H). [20]

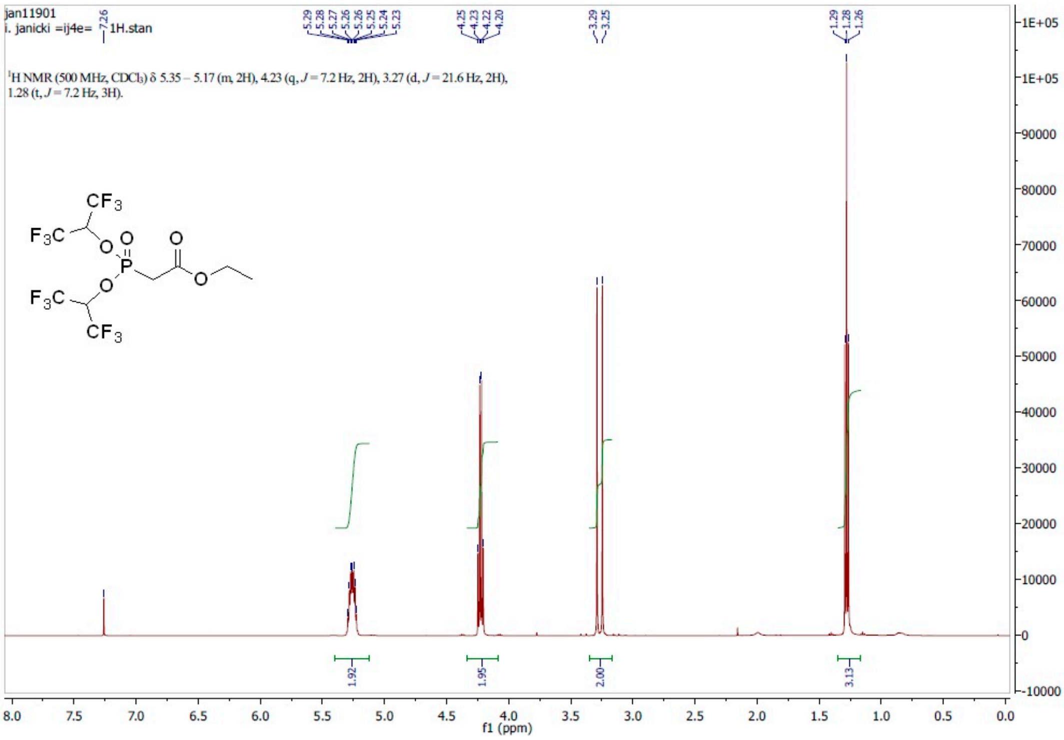

^{31}P NMR ($CDCl_3$, 202 MHz): δ = 26.25. [20]

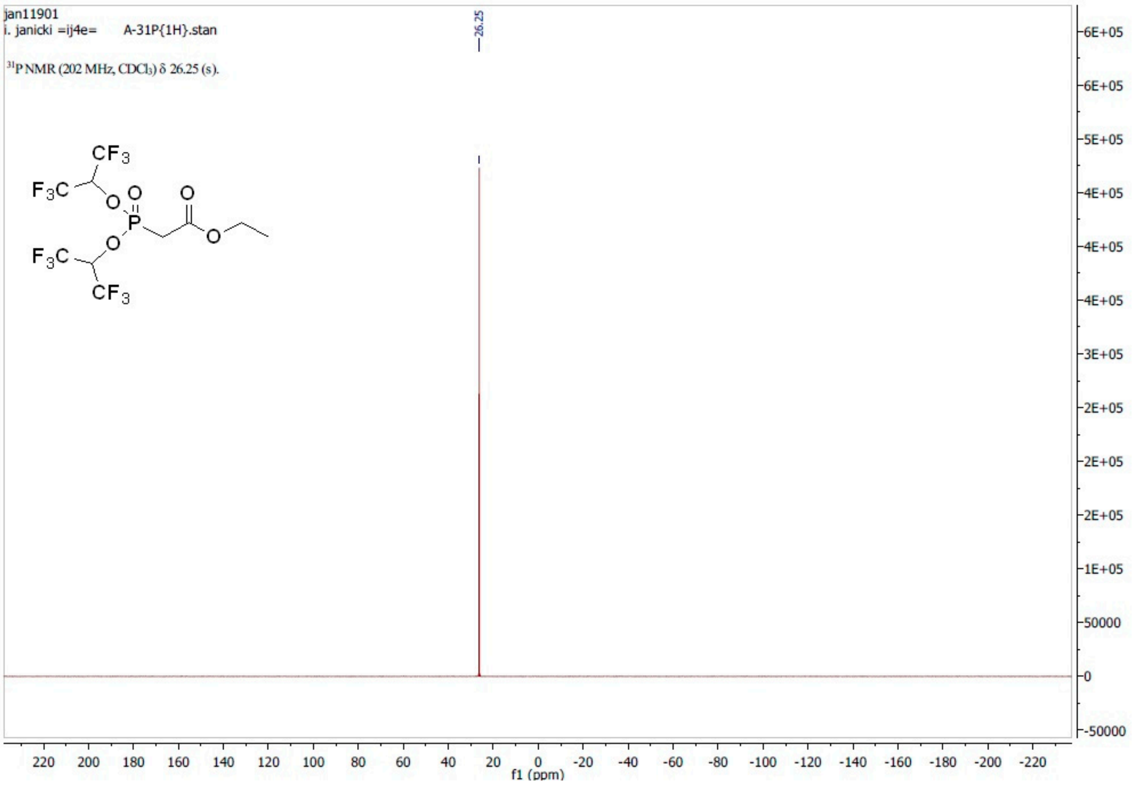

Z-3aa

^{1}H NMR (400 MHz, Chloroform-*d*) δ 7.64 – 7.51 (m, 2H), 7.44 – 7.29 (m, 3H), 6.96 (d, *J* = 12.6 Hz, 1H), 5.95 (d, *J* = 12.6 Hz, 1H), 3.71 (s, 3H). [24] 97:3 *Z*:*E*.

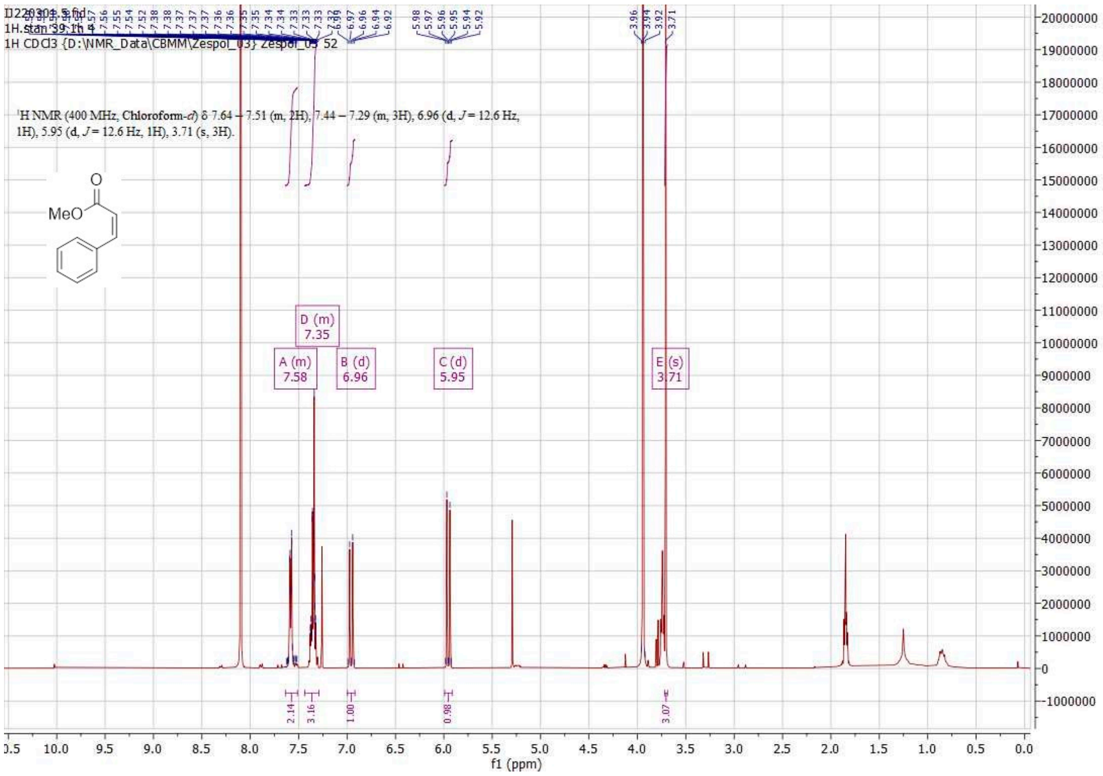

Z-3ab

^{1}H NMR (400 MHz, Chloroform-*d*) δ 7.54 (d, J = 8.2 Hz, 2H), 7.18 (d, J = 8.0 Hz, 2H), 6.93 (d, J = 12.7 Hz, 1H), 5.91 (d, J = 12.6 Hz, 1H), 3.73 (s, 3H), 2.38 (s, 3H). [24] 96:4 *Z*:*E*.

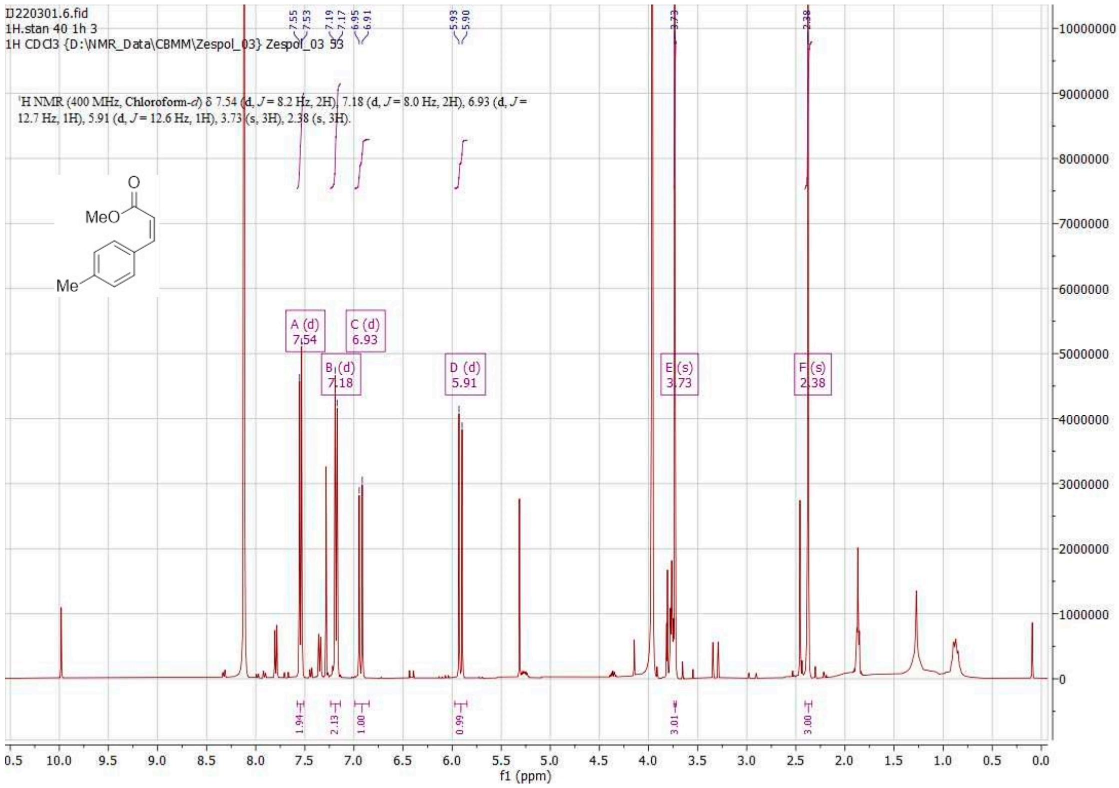

Z-3ac

^{1}H NMR (400 MHz, Chloroform-*d*) δ 7.47 – 7.11 (m, 4H), 6.92 (d, J = 12.6 Hz, 1H), 5.93 (d, J = 12.6 Hz, 1H), 3.70 (s, 3H), 2.35 (s, 3H). [25] 95:5 *Z*:*E*.

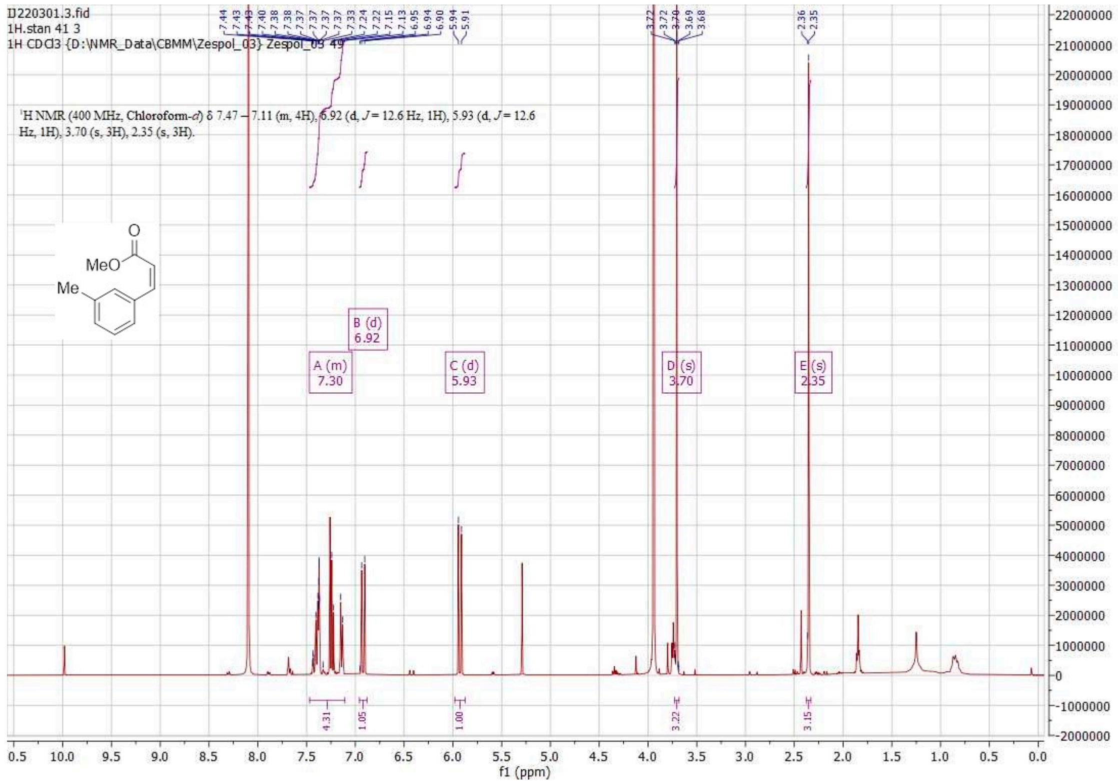

Z-3ad

^{1}H NMR (400 MHz, Chloroform-*d*) δ 7.31 (d, *J* = 7.7 Hz, 1H), 7.25 – 7.07 (m, 4H), 6.03 (d, *J* = 12.2 Hz, 1H), 3.63 (s, 3H), 2.28 (s, 3H). [26] 97:3 *Z*:*E*.

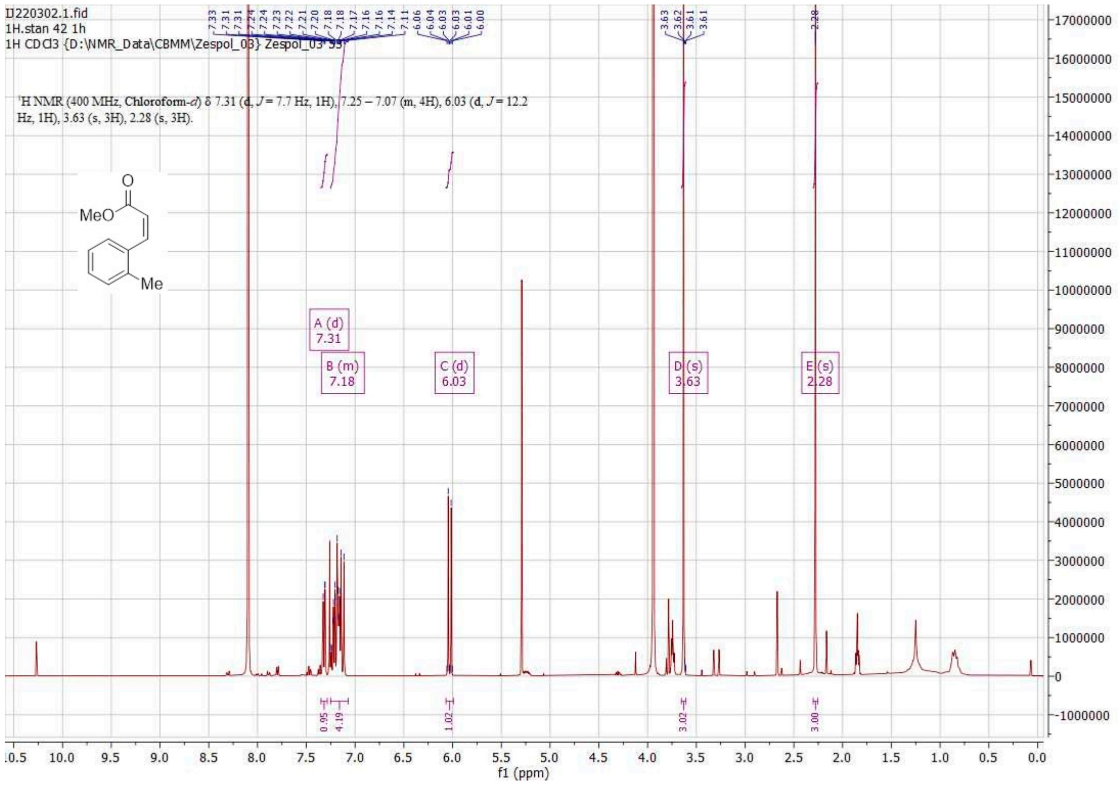

Z-3ae

^{1}H NMR (400 MHz, Chloroform-*d*) δ 7.57 (d, *J* = 8.2 Hz, 2H), 7.34 (d, *J* = 8.3 Hz, 2H), 6.91 (d, *J* = 12.6 Hz, 1H), 5.98 (d, *J* = 12.7 Hz, 1H), 3.73 (s, 3H). [24] 95:5 *Z*:*E*.

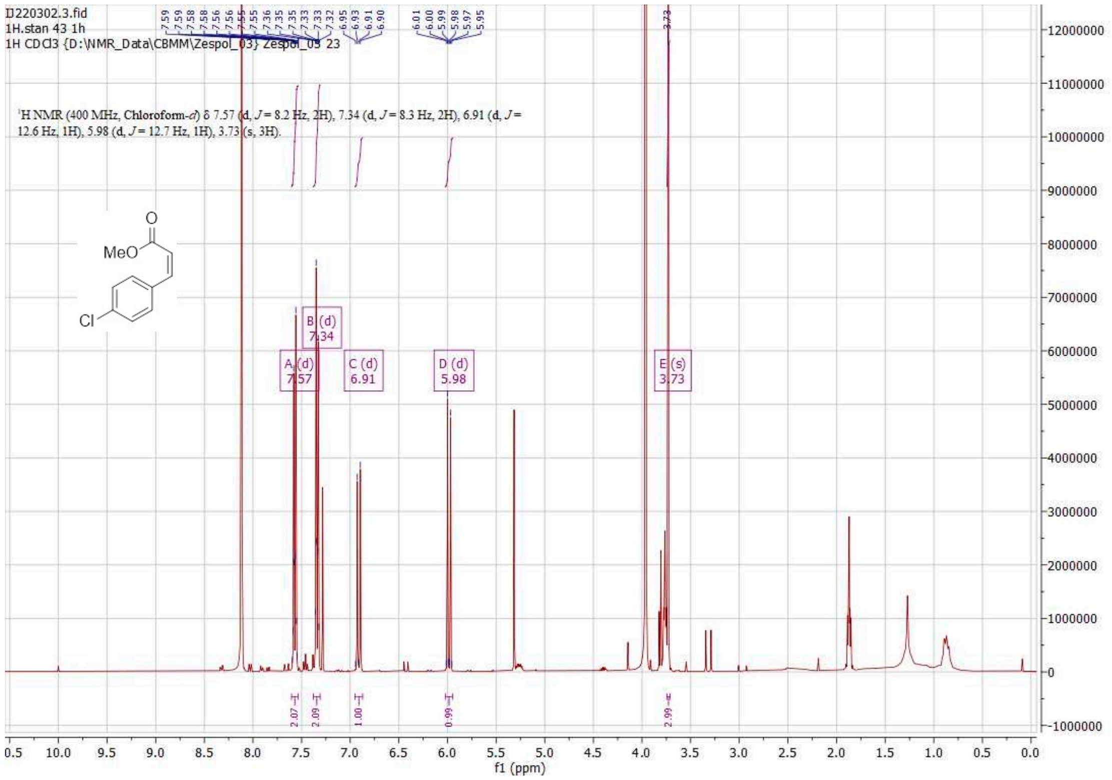

Z-3af

^{1}H NMR (400 MHz, Chloroform-*d*) δ 7.47 (s, 4H), 6.86 (d, *J* = 12.7 Hz, 1H), 5.97 (d, *J* = 12.6 Hz, 1H), 3.70 (s, 3H). [24] 95:5 *Z*:*E*.

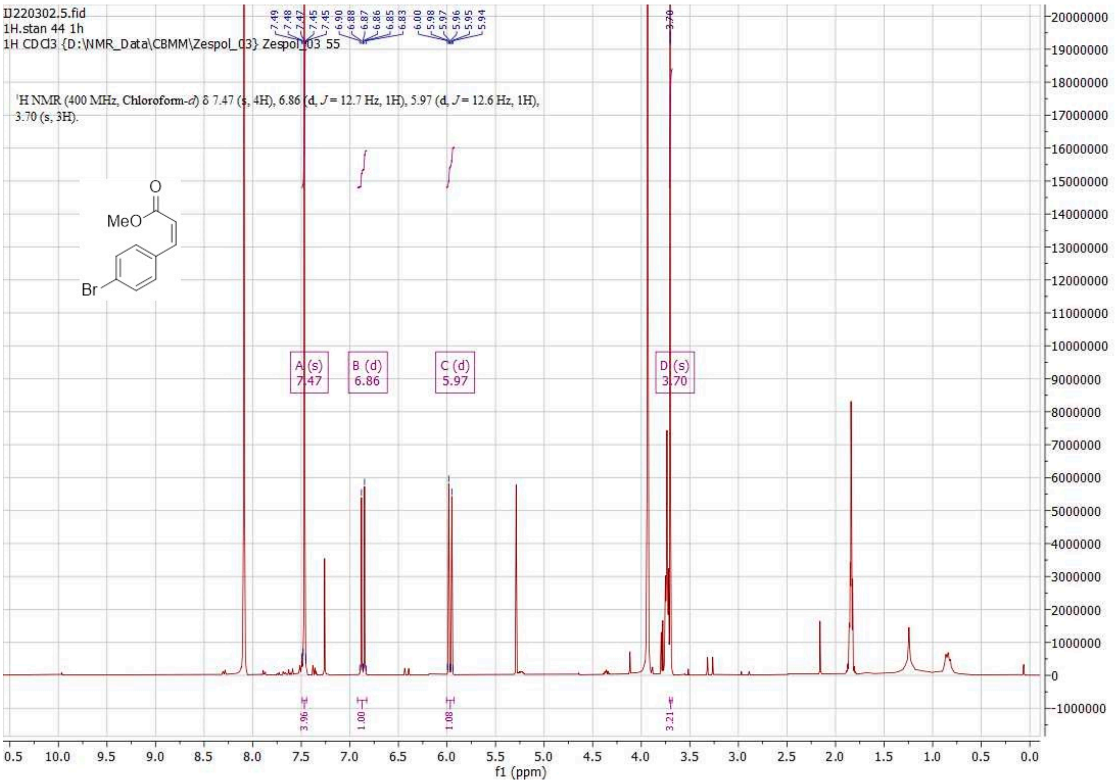

Z-3ag

^{1}H NMR (400 MHz, Chloroform-*d*) δ 8.22 (d, *J* = 8.9 Hz, 2H), 7.69 (d, *J* = 8.6 Hz, 2H), 7.04 (d, *J* = 12.5 Hz, 1H), 6.15 (d, *J* = 12.5 Hz, 1H), 3.73 (s, 3H). [24] 94:6 *Z*:*E*.

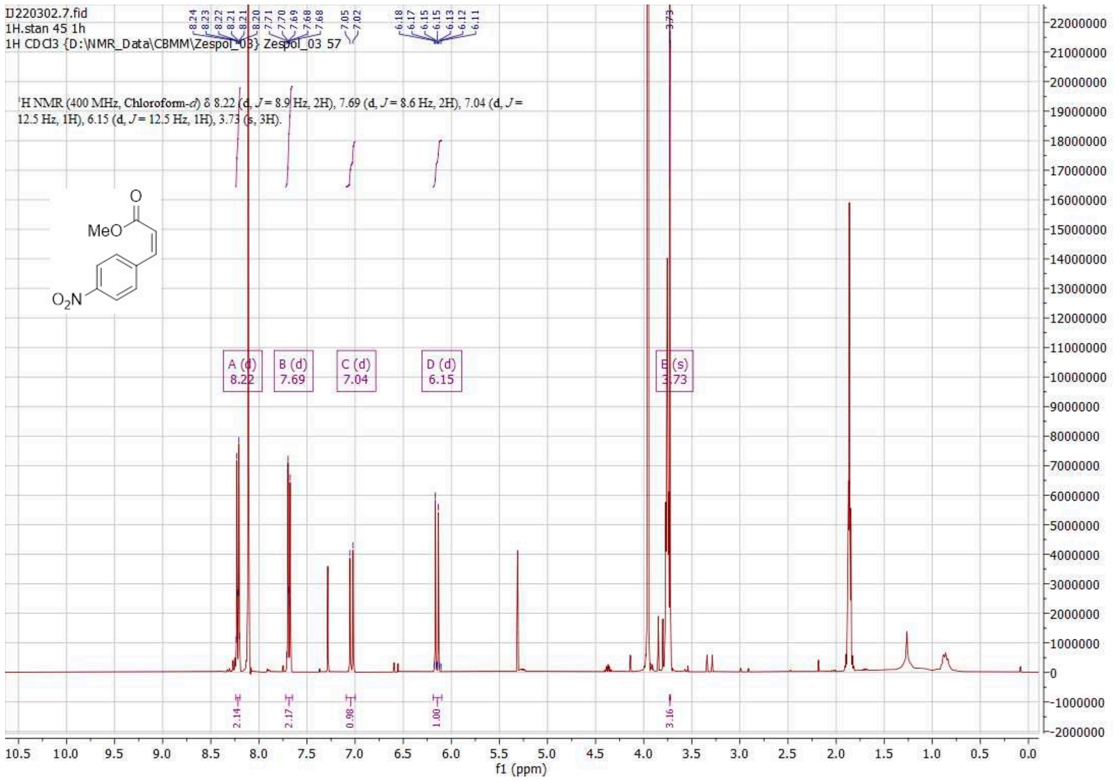

***Z*-3ah**

^{1}H NMR (400 MHz, Chloroform-*d*) δ 7.68 (d, *J* = 3.6 Hz, 1H), 7.47 (d, *J* = 1.7 Hz, 1H), 6.79 (d, *J* = 12.9 Hz, 1H), 6.50 (ddd, *J* = 3.5, 1.7, 0.7 Hz, 1H), 5.73 (d, *J* = 12.9 Hz, 1H), 3.75 (s, 3H). [27] 92:8 *Z*:*E*.

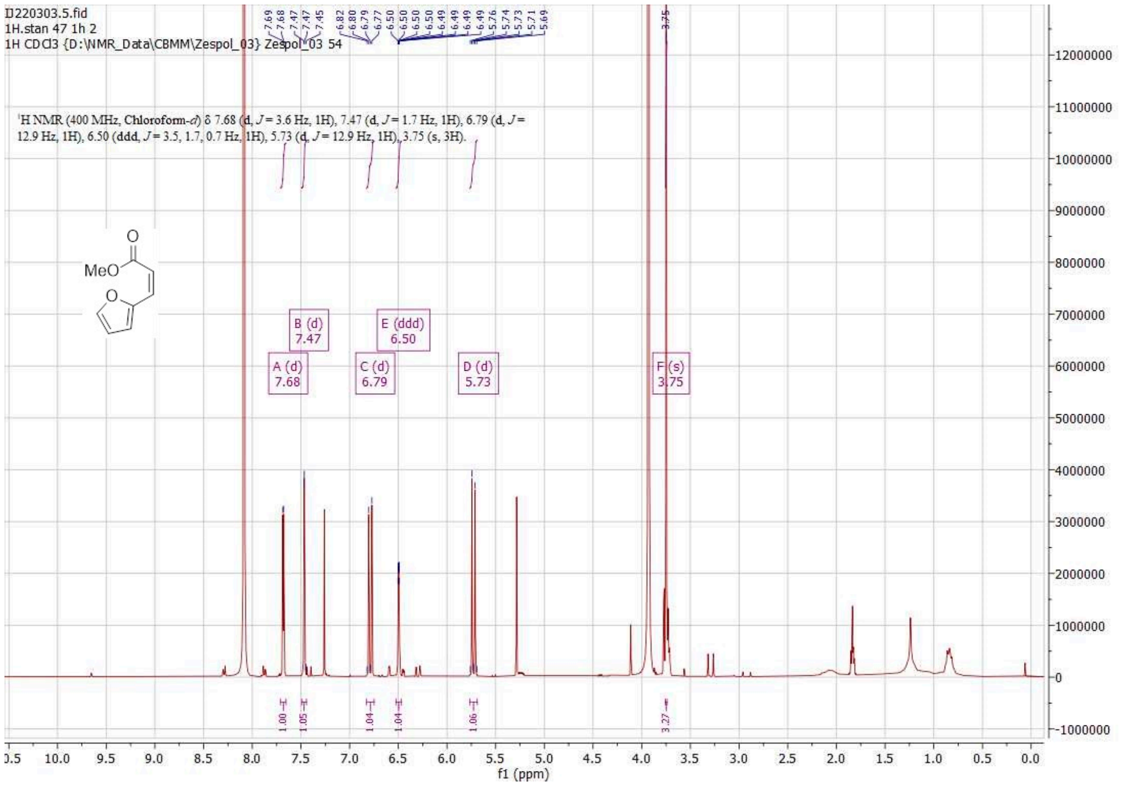

***Z*-3ai**

^{1}H NMR (400 MHz, Chloroform-*d*) δ 7.52 (d, *J* = 5.1 Hz, 1H), 7.44 (d, *J* = 3.1 Hz, 1H), 7.10 (d, *J* = 12.5 Hz, 1H), 7.05 (dd, *J* = 5.2, 3.7 Hz, 1H), 5.74 (d, *J* = 12.5 Hz, 1H), 3.78 (s, 3H). [28] 94:6 *Z*:*E*.

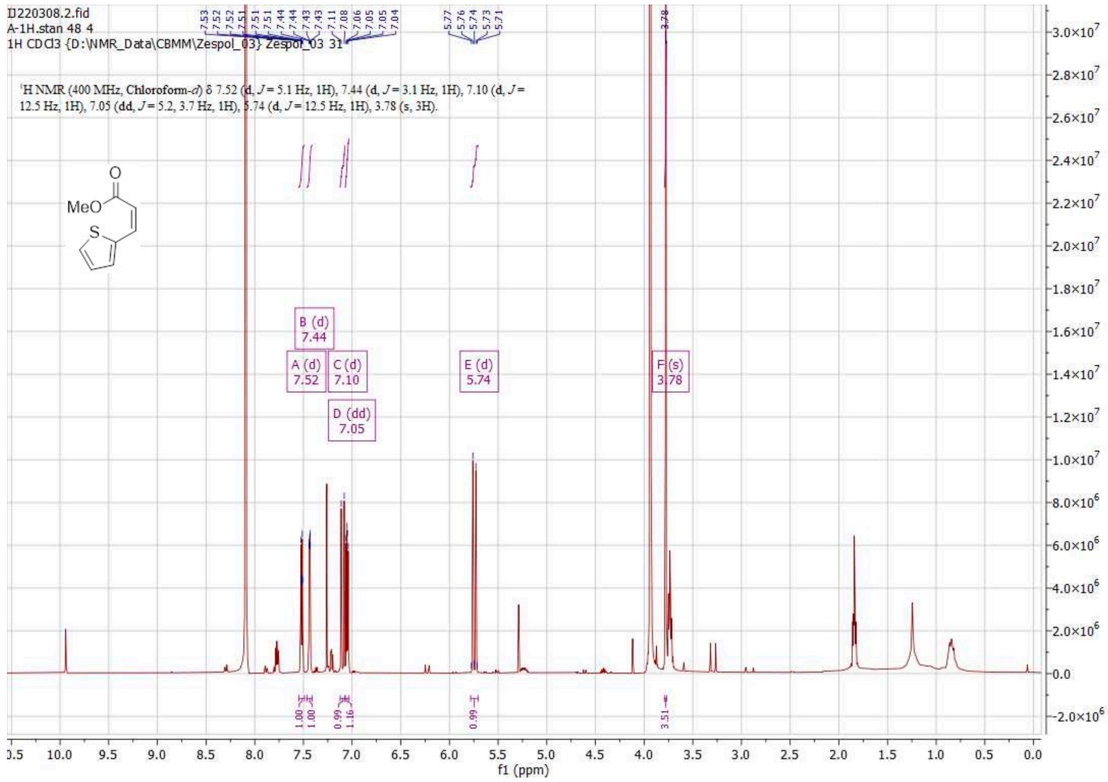

***Z*-3aj**

^{1}H NMR (400 MHz, Chloroform-*d*) δ 8.18 – 8.11 (m, 1H), 7.60 – 7.49 (m, 2H), 7.48 – 7.27 (m, 3H), 6.92 – 6.68 (m, 2H), 5.73 (d, *J* = 10.9 Hz, 1H), 3.76 (s, 3H). [29] 89:11 *Z*:*E*.

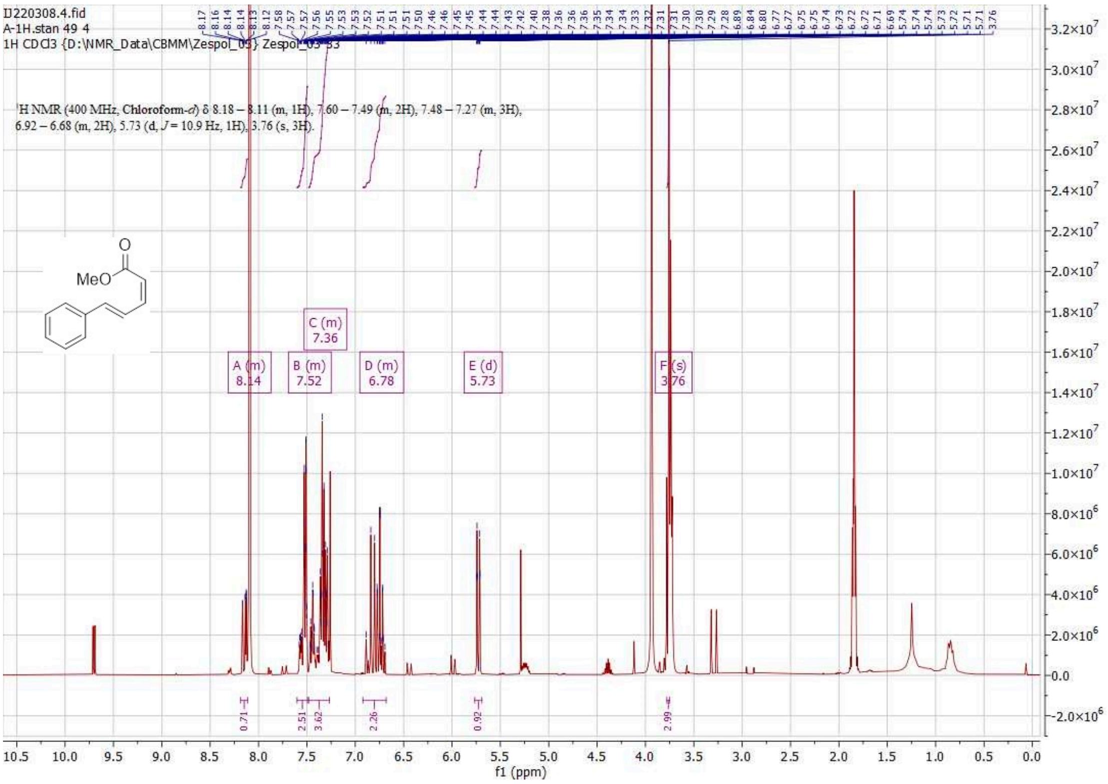

***Z*-3ak**

^{1}H NMR (400 MHz, Chloroform-*d*) δ 6.03 (dd, *J* = 11.5, 9.9 Hz, 1H), 5.65 (dd, *J* = 11.5, 1.0 Hz, 1H), 3.69 (s, 3H), 3.35 – 3.22 (m, 1H), 1.74 – 1.62 (m, 5H), 1.42 – 1.00 (m, 5H). [30] 86:14 *Z*:*E*.

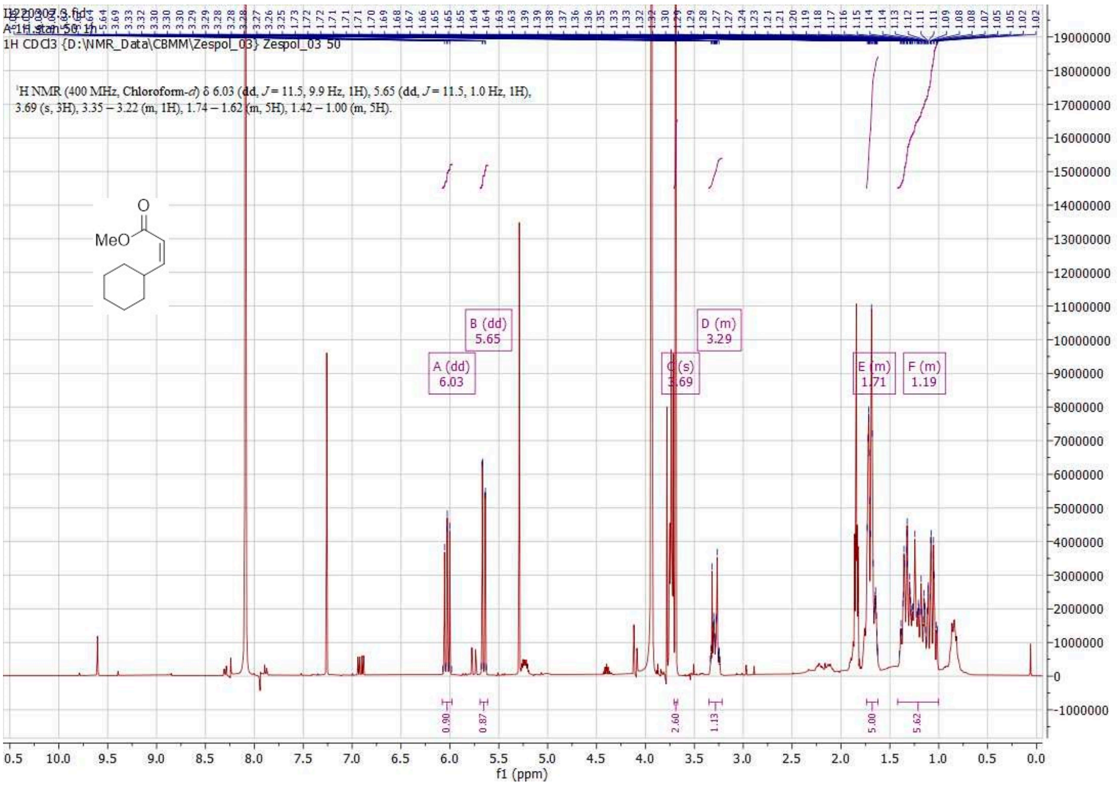

Z-3al

^{1}H NMR (400 MHz, Chloroform-*d*) δ 6.25 (dt, *J* = 11.5, 7.5 Hz, 1H), 5.78 (dt, *J* = 11.5, 1.7 Hz, 1H), 3.72 (s, 3H), 2.66 (qd, *J* = 7.5, 1.8 Hz, 2H), 1.49 – 1.39 (m, 2H), 1.38 – 1.23 (m, 8H), 0.89 (t, *J* = 6.7 Hz, 3H). [31] 88:12 *Z*:*E*.

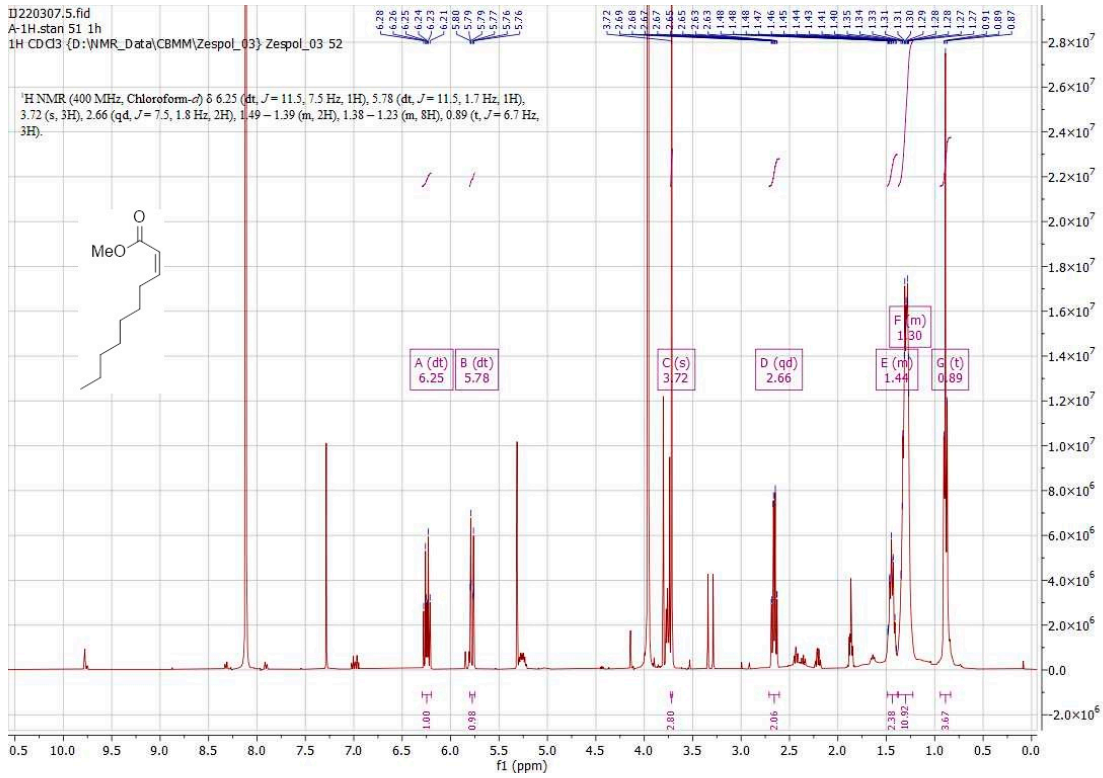

Z-3am

^{1}H NMR (400 MHz, Chloroform-*d*) δ 6.23 (dt, *J* = 11.5, 7.5 Hz, 1H), 5.76 (dt, *J* = 11.5, 1.8 Hz, 1H), 3.69 (s, 3H), 2.63 (qd, *J* = 7.5, 1.7 Hz, 2H), 1.50 – 1.38 (m, 2H), 1.36 – 1.18 (m, 4H), 0.87 (t, *J* = 6.3 Hz, 3H). [32] 88:12 *Z*:*E*.

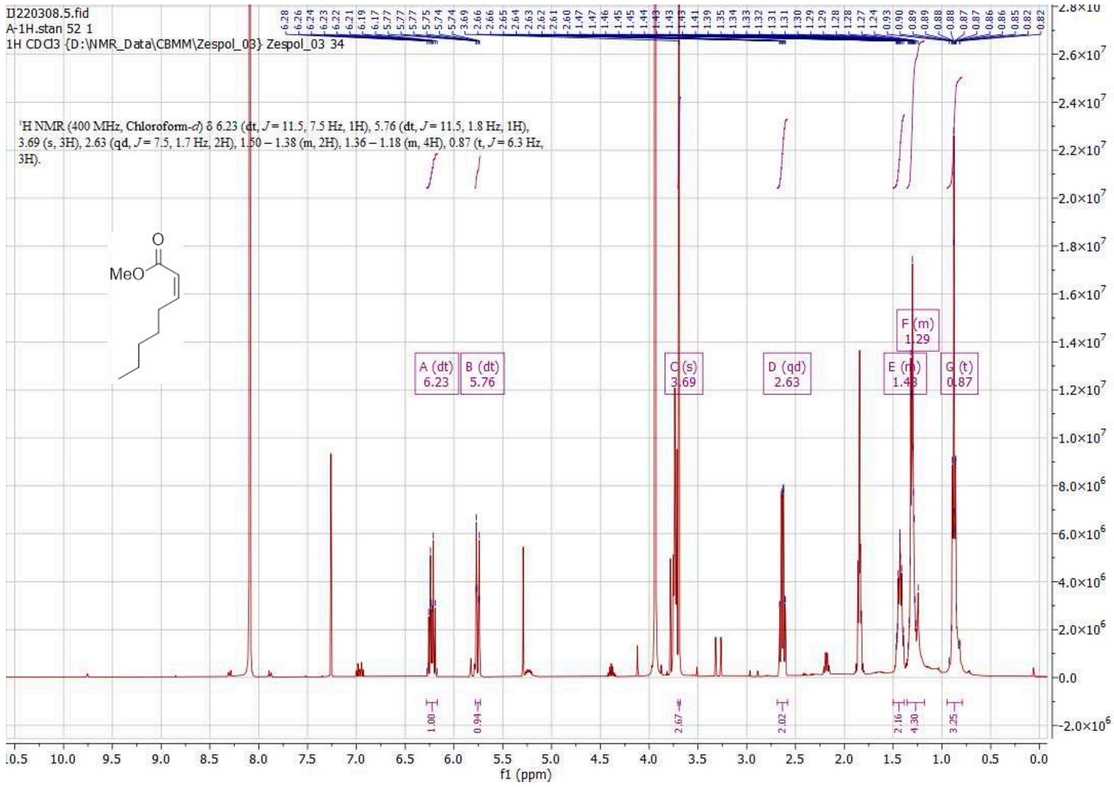

***Z*-3ba**

^{1}H NMR (400 MHz, Chloroform-*d*) δ 7.60 – 7.53 (m, 2H), 7.38 – 7.29 (m, 3H), 6.94 (d, *J* = 12.5 Hz, 1H), 5.94 (d, *J* = 12.6 Hz, 1H), 4.16 (q, *J* = 7.1 Hz, 2H), 1.23 (t, *J* = 7.1 Hz, 3H). [24] 97:3 *Z*:*E*.

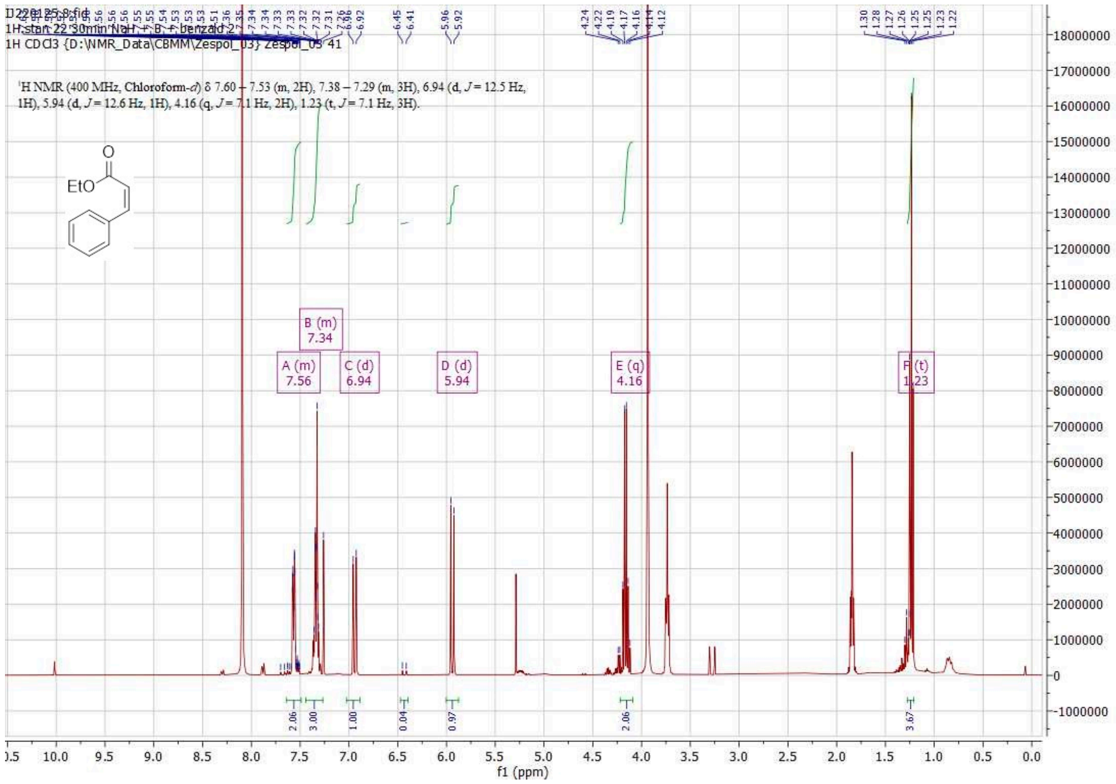

***Z*-3bb**

^{1}H NMR (400 MHz, Chloroform-*d*) δ 7.51 (d, *J* = 8.1 Hz, 2H), 7.15 (d, *J* = 7.9 Hz, 2H), 6.89 (d, *J* = 12.6 Hz, 1H), 5.88 (d, *J* = 12.7 Hz, 1H), 4.17 (q, *J* = 7.1 Hz, 2H), 2.35 (s, 3H), 1.25 (t, *J* = 7.1 Hz, 3H). [25] 96:4 *Z*:*E*.

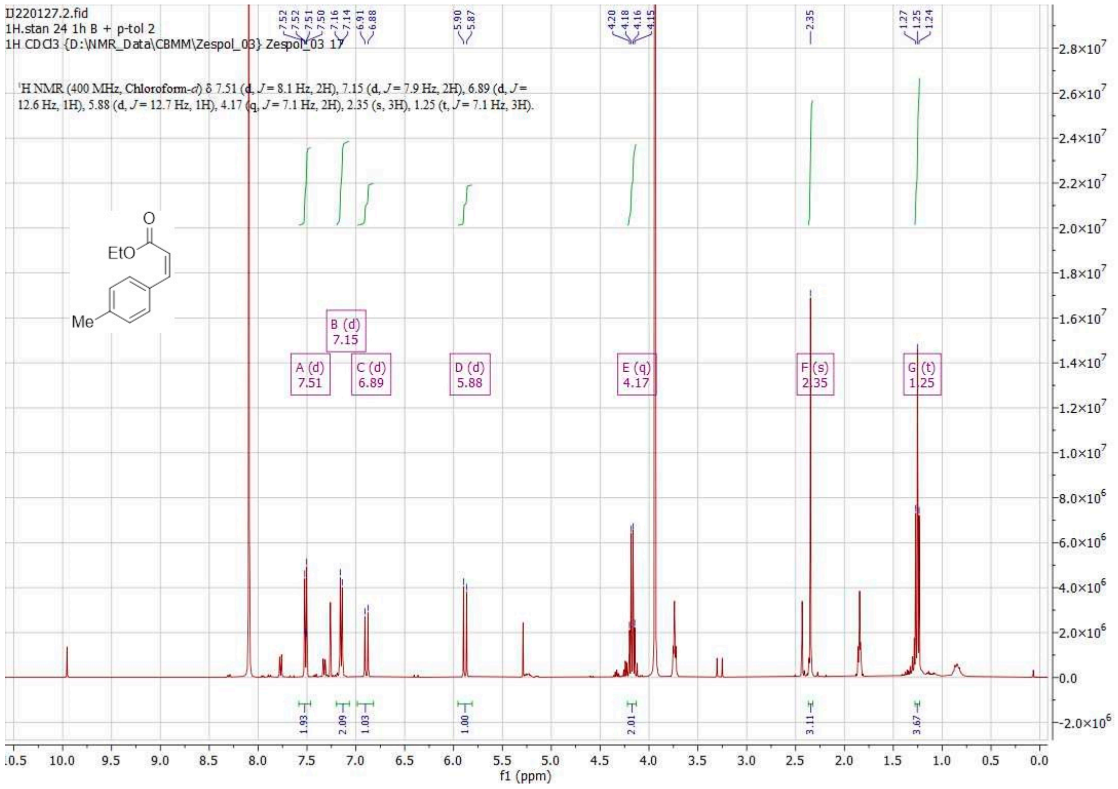

***Z*-3bc**

^{1}H NMR (400 MHz, Chloroform-*d*) δ 7.37 (d, *J* = 11.2 Hz, 2H), 7.23 (t, *J* = 7.7 Hz, 1H), 7.13 (d, *J* = 7.6 Hz, 1H), 6.91 (d, *J* = 12.6 Hz, 1H), 5.92 (d, *J* = 12.6 Hz, 1H), 4.17 (q, *J* = 7.1 Hz, 2H), 2.35 (s, 3H), 1.24 (t, *J* = 7.1 Hz, 3H). [33] 96:4 *Z*:*E*.

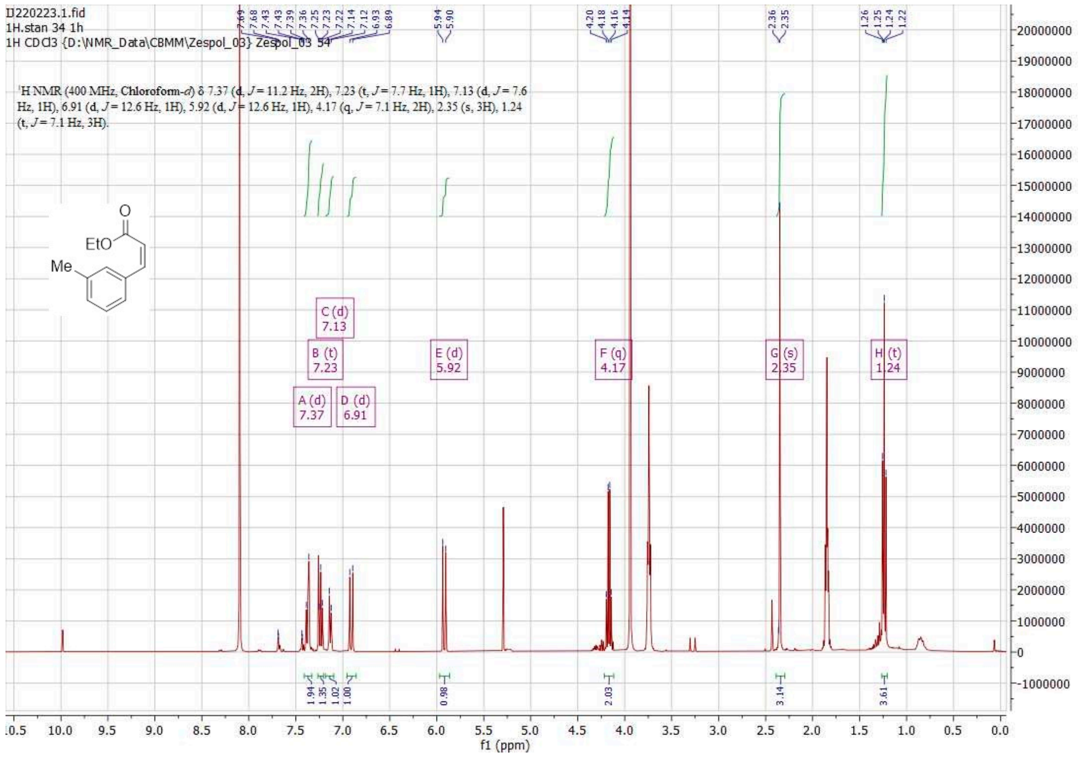

***Z*-3bd**

^{1}H NMR (400 MHz, Chloroform-*d*) δ 7.31 (d, *J* = 8.2 Hz, 1H), 7.24 – 7.06 (m, 4H), 6.02 (d, *J* = 12.2 Hz, 1H), 4.08 (q, *J* = 7.1 Hz, 2H), 2.28 (s, 3H), 1.14 (t, *J* = 7.1 Hz, 3H). [33] 98:2 *Z*:*E*.

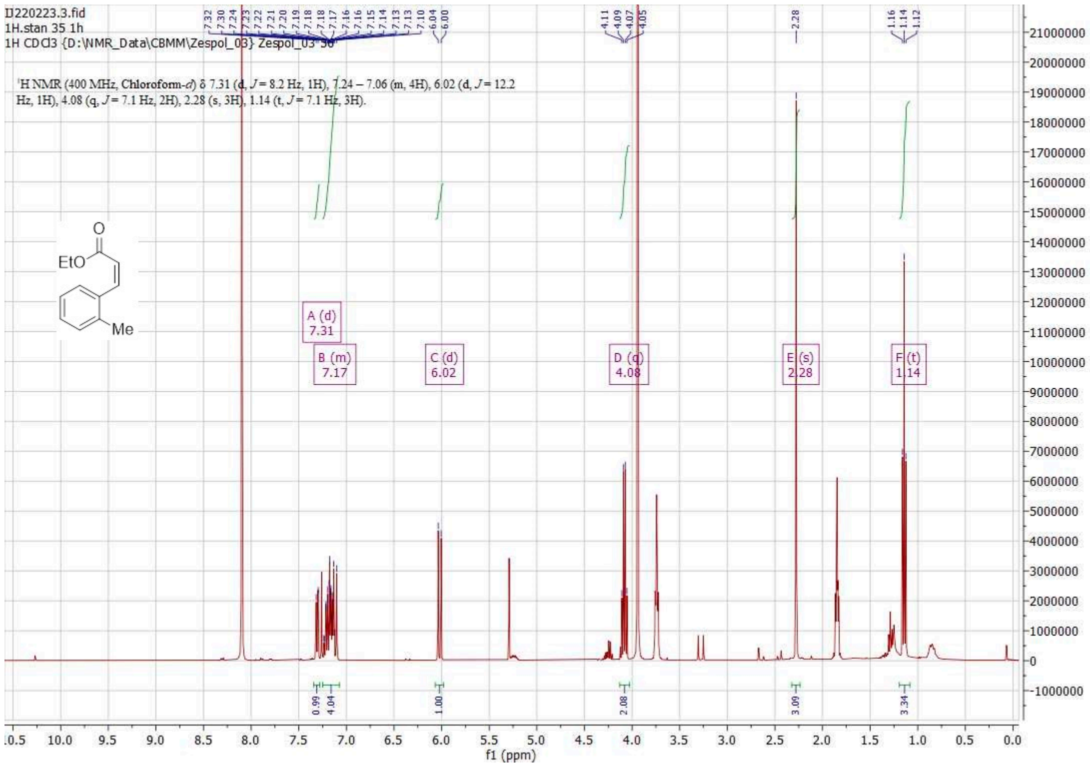

***Z*-3be**

^{1}H NMR (400 MHz, Chloroform-*d*) δ 7.54 (d, *J* = 8.6 Hz, 2H), 7.31 (d, *J* = 8.5 Hz, 2H), 6.87 (d, *J* = 12.6 Hz, 1H), 5.95 (d, *J* = 12.6 Hz, 1H), 4.17 (q, *J* = 7.1 Hz, 2H), 1.25 (t, *J* = 7.1 Hz, 3H). [33] 96:4 *Z*:*E*.

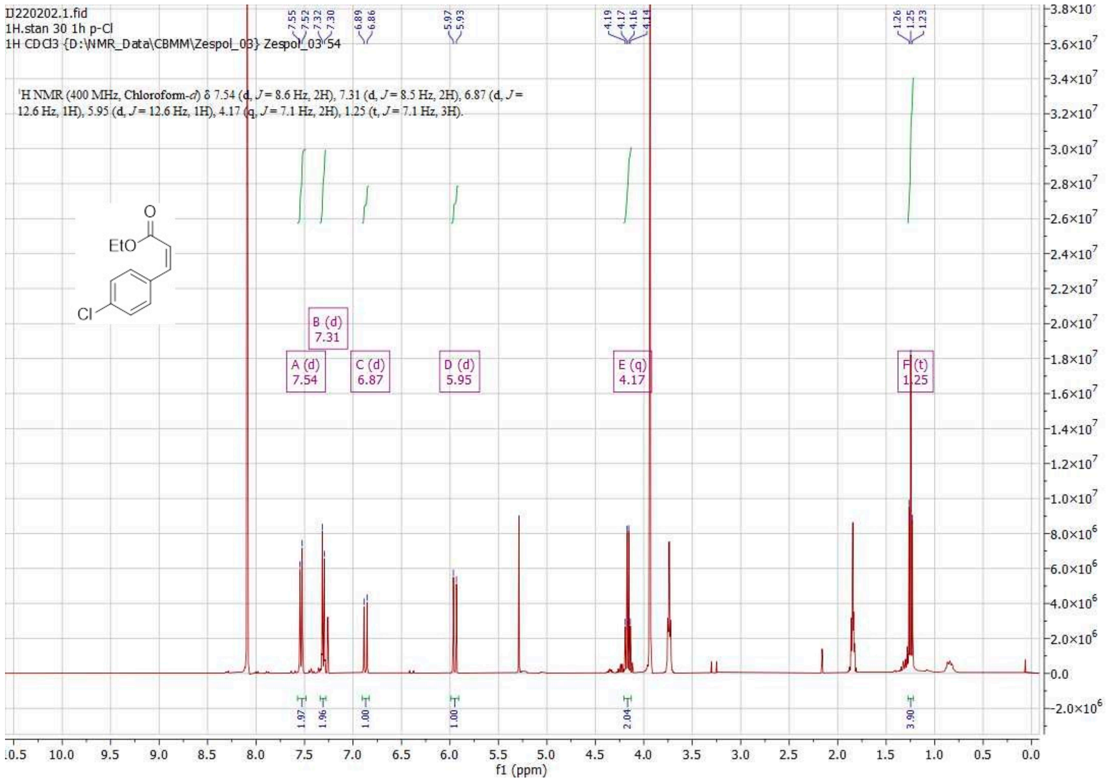

***Z*-3bf**

^{1}H NMR (400 MHz, Chloroform-*d*) δ 7.49 (s, 4H), 6.88 (d, *J* = 12.6 Hz, 1H), 5.98 (d, *J* = 12.6 Hz, 1H), 4.19 (q, *J* = 7.1 Hz, 2H), 1.27 (t, *J* = 7.1 Hz, 3H). [33] 94:6 *Z*:*E*.

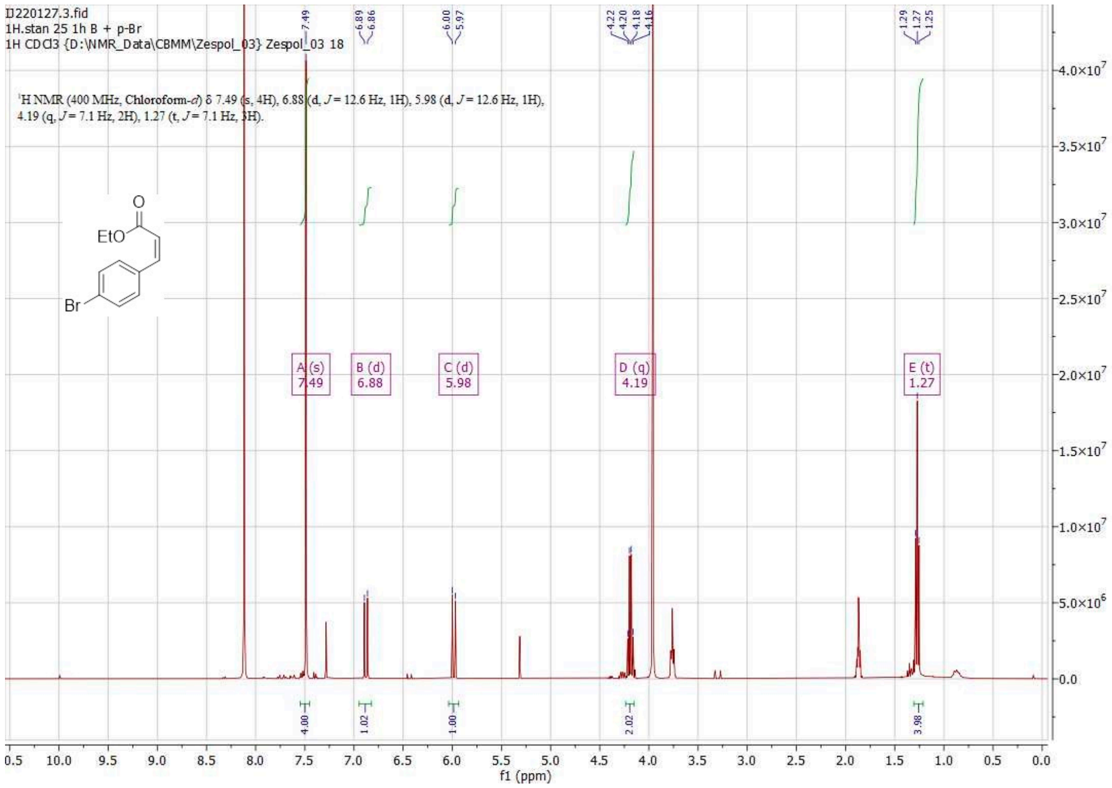

Z-3bg

^{1}H NMR (400 MHz, Chloroform-*d*) δ 8.19 (d, *J* = 8.8 Hz, 2H), 7.66 (d, *J* = 8.6 Hz, 2H), 7.00 (d, *J* = 12.5 Hz, 1H), 6.12 (d, *J* = 12.5 Hz, 1H), 4.16 (q, *J* = 7.1 Hz, 2H), 1.23 (t, *J* = 7.1 Hz, 3H). [33] 95:5 *Z*:*E*.

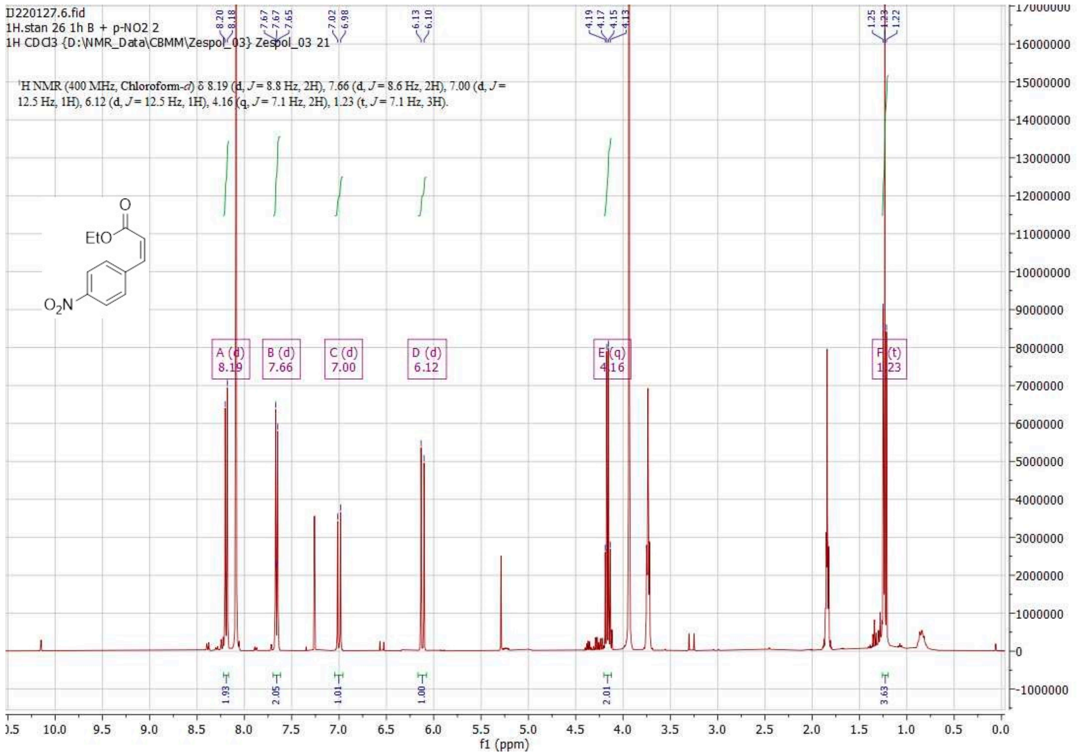

Z-3bh

^{1}H NMR (400 MHz, Chloroform-*d*) δ 7.66 (d, *J* = 3.5 Hz, 1H), 7.46 (d, *J* = 1.8 Hz, 1H), 6.78 (d, *J* = 12.9 Hz, 1H), 6.49 (ddd, *J* = 3.6, 1.8, 0.7 Hz, 1H), 5.72 (d, *J* = 12.9 Hz, 1H), 4.21 (q, *J* = 7.1 Hz, 2H), 1.31 (t, *J* = 7.1 Hz, 3H). [33] 95:5 *Z*:*E*.

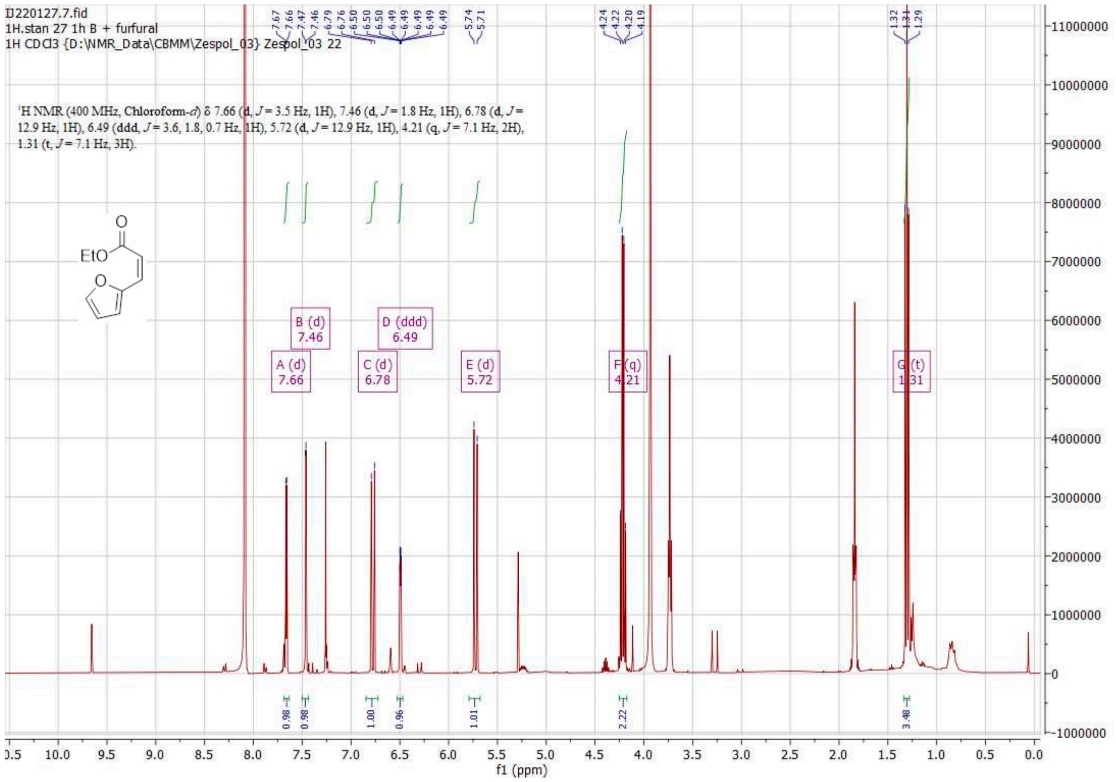

***Z*-3bi**

^{1}H NMR (400 MHz, Chloroform-*d*) δ 7.50 (d, *J* = 5.1 Hz, 1H), 7.42 (d, *J* = 3.7 Hz, 1H), 7.07 (d, *J* = 12.1 Hz, 1H), 7.03 (dd, *J* = 5.2, 3.7 Hz, 1H), 5.72 (d, *J* = 12.5 Hz, 1H), 4.23 (q, *J* = 7.1 Hz, 2H), 1.31 (t, *J* = 7.1 Hz, 3H). [34] 95:5 *Z*:*E*.

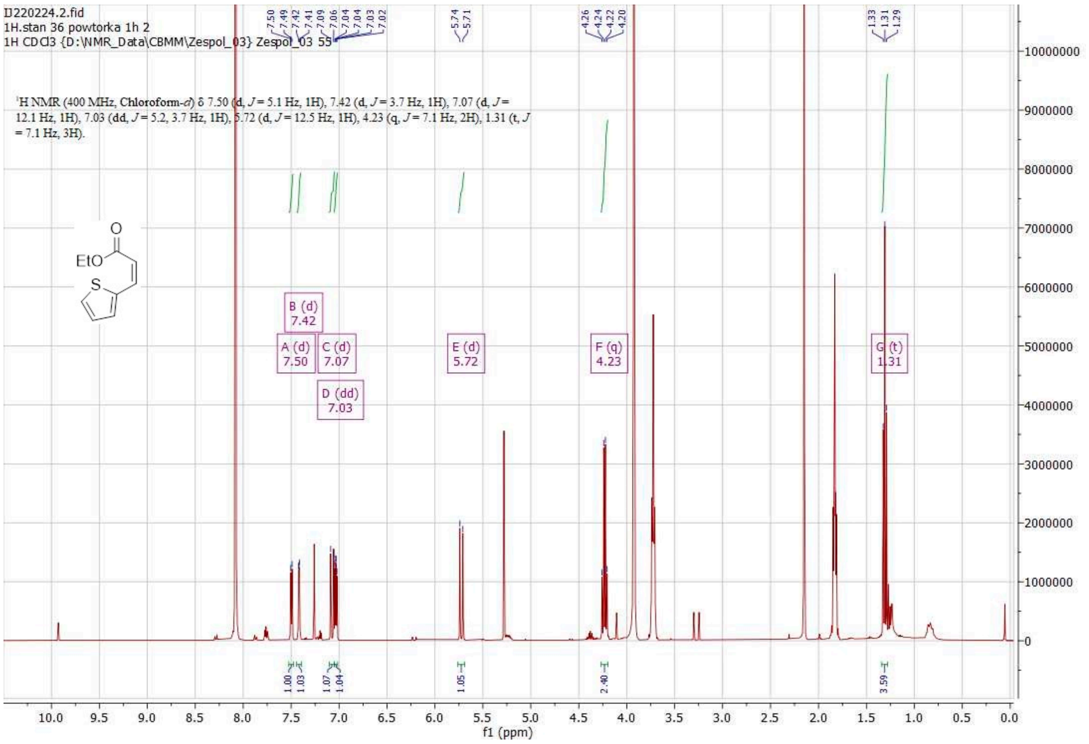

***Z*-3bj**

^{1}H NMR (400 MHz, Chloroform-*d*) δ 8.14 (dd, *J* = 16.1, 11.7 Hz, 1H), 7.51 (d, *J* = 7.9 Hz, 2H), 7.37 – 7.27 (m, 3H), 6.81 (d, *J* = 15.7 Hz, 1H), 6.72 (d, *J* = 11.4 Hz, 1H), 5.72 (d, *J* = 11.2 Hz, 1H), 4.22 (q, *J* = 7.1 Hz, 2H), 1.32 (t, *J* = 7.1 Hz, 3H). [35] 91:9 *Z*:*E*.

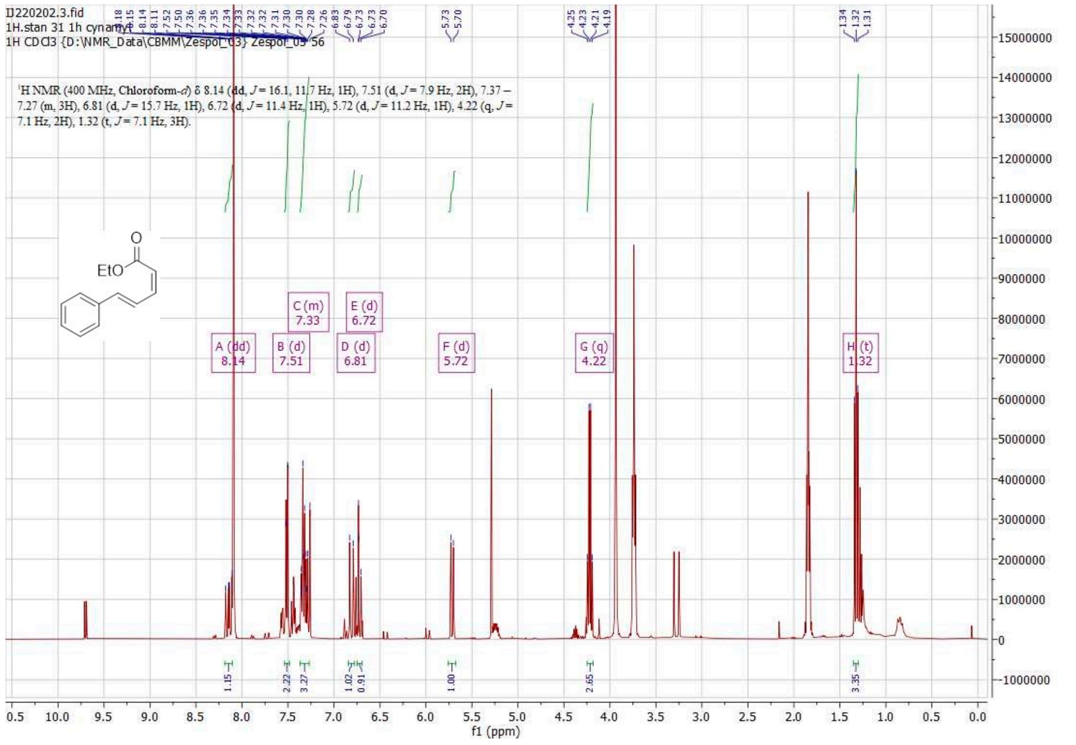

Z-3bk

^{1}H NMR (400 MHz, Chloroform-*d*) δ 6.01 (dd, *J* = 11.5, 9.8 Hz, 1H), 5.64 (dd, *J* = 11.5, 1.1 Hz, 1H), 4.15 (q, *J* = 7.1 Hz, 2H), 3.34 – 3.21 (m, 1H), 1.78 – 1.66 (m, 5H), 1.40 – 1.29 (m, 2H), 1.28 (t, *J* = 7.2 Hz, 3H), 1.27 – 0.99 (m, 3H). [36] 87:13 *Z*:*E*.

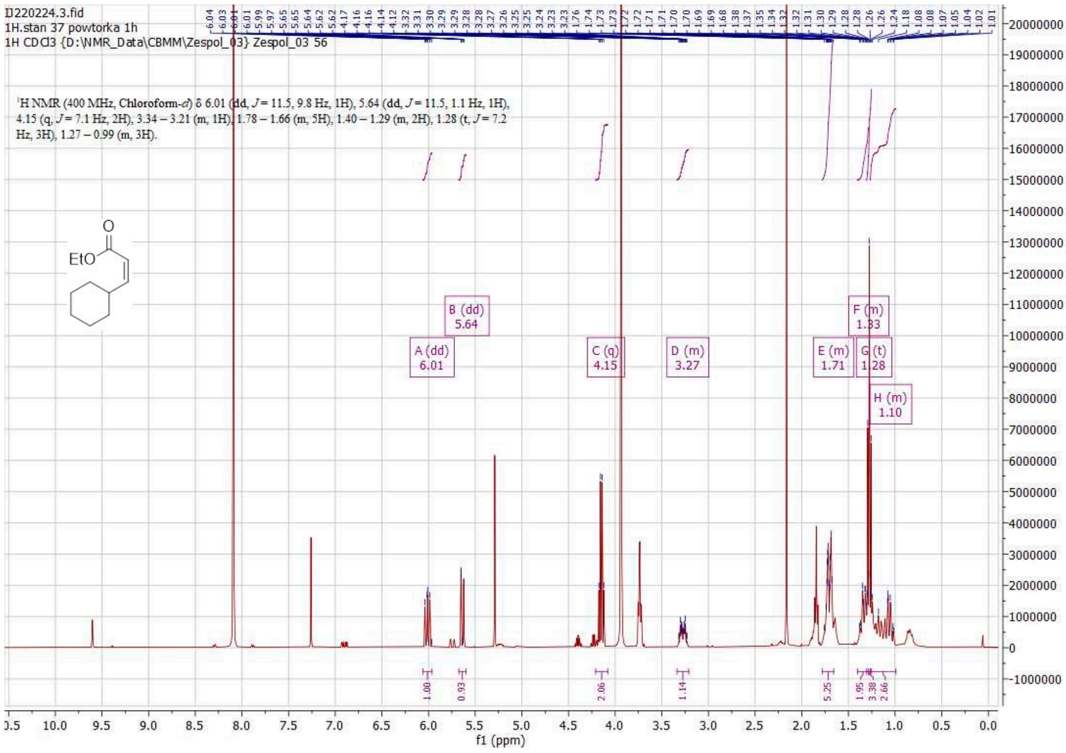

Z-3bl

^{1}H NMR (400 MHz, Chloroform-*d*) δ 6.20 (dt, *J* = 11.5, 7.5 Hz, 1H), 5.74 (dt, *J* = 11.5, 1.8 Hz, 1H), 4.15 (q, *J* = 7.1 Hz, 2H), 2.63 (qd, *J* = 7.5, 1.7 Hz, 2H), 1.42 (dq, *J* = 14.5, 7.2 Hz, 2H), 1.31 – 1.22 (m, 11H), 0.86 (t, *J* = 6.8 Hz, 3H) [15] 88:12 *Z*:*E*.

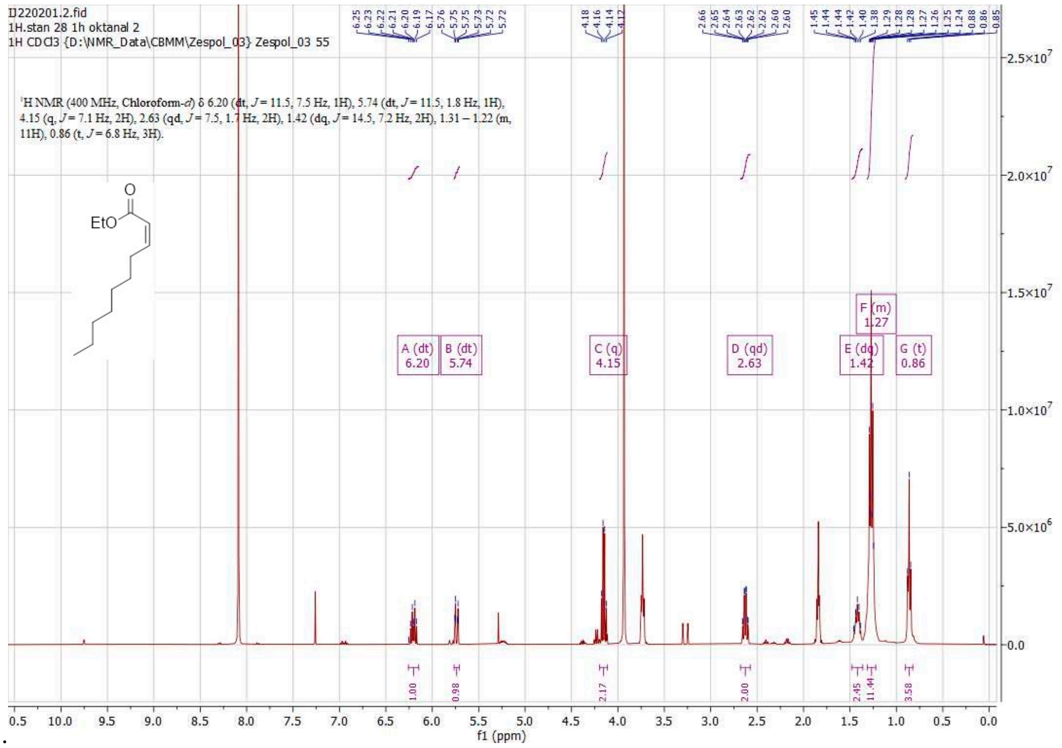

.

***Z*-3bm**

^{1}H NMR (400 MHz, Chloroform-*d*) δ 6.23 (dt, *J* = 11.6, 7.5 Hz, 1H), 5.77 (dt, *J* = 11.5, 1.7 Hz, 1H), 4.18 (q, *J* = 7.1 Hz, 2H), 2.65 (qd, *J* = 7.6, 1.7 Hz, 2H), 1.51 – 1.41 (m, 2H), 1.37 – 1.25 (m, 7H), 0.90 (t, *J* = 6.7 Hz, 3H). [38] 88:12 *Z*:*E*.

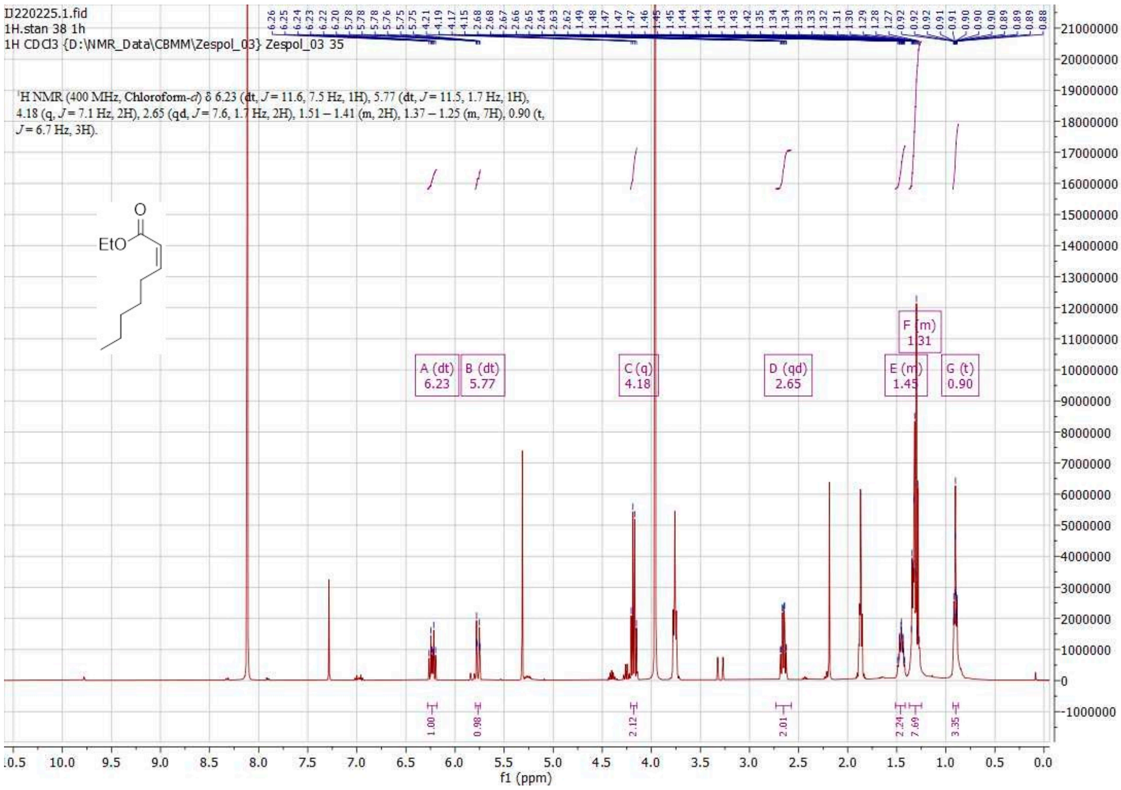

Supplement: Supplementary file 1 [file molecules-27-07138-s001.zip › molecules-1947449-supplementary.pdf]
